# Deletion of SM22α disrupts the structure and function of caveolae and T-tubules in cardiomyocytes, contributing to heart failure

**DOI:** 10.1371/journal.pone.0271578

**Published:** 2022-07-18

**Authors:** Jun Wu, Wei Wang, Yaomeng Huang, Haochen Wu, Jiabin Wang, Mei Han

**Affiliations:** 1 Department of Biochemistry and Molecular Biology, College of Basic Medicine, Key Laboratory of Medical Biotechnology of Hebei Province, Key Laboratory of Neural and Vascular Biology, Ministry of Education, Hebei Medical University, Shijiazhuang, China; 2 Department of Physiology, College of Basic Medicine, Hebei Medical University, Shijiazhuang, China; University of Debrecen, HUNGARY

## Abstract

**Aims:**

Smooth muscle 22-alpha (SM22α) is an actin-binding protein that plays critical roles in mediating polymerization of actin filaments and stretch sensitivity of cytoskeleton in vascular smooth muscle cells (VSMCs). Multiple lines of evidence indicate the existence of SM22α in cardiomyocytes. Here, we investigated the effect of cardiac SM22α on the membrane architecture and functions of cardiomyocytes to pressure overload.

**Methods:**

SM22α knock-out (KO) mice were utilized to assess the role of SM22α in the heart. Echocardiography was used to evaluate cardiac function, transverse aortic constriction (TAC) was used to induce heart failure, cell shortening properties were measured by IonOptix devices in intact cardiomyocytes, Ca^2+^ sensitivity of myofilaments was measured in permeabilized cardiomyocytes. Confocal microscopy, electron microscopy, western blotting, co-immunoprecipitation (co-IP), Real-Time Quantitative Reverse Transcription PCR (qRT-PCR) techniques were used to perform functional and structural analysis.

**Results:**

SM22α ablation did not alter cardiac function at baseline, but mRNA levels of atrial natriuretic peptide (ANP), brain natriuretic peptide (BNP) and β-myosin heavy chain (β-MHC) were increased significantly compared with wild type (WT) controls. The membrane architecture was severely disrupted in SM22α KO cardiomyocytes, with disassembly and flattening of caveolae and disrupted T-tubules. Furthermore, SM22α was co-immunoprecipitated with caveolin-3 (Cav3), and the interaction between Cav3 and actin was significantly reduced in SM22α KO cells. SM22α KO cardiomyocytes displayed asynchronized SR Ca^2+^ release, significantly increased Ca^2+^ spark frequency. Additionally, the kinetics of sarcomere shortening was abnormal, accompanied with increased sensitivity and reduced maximum response of myofilaments to Ca^2+^ in SM22α KO cardiomyocytes. SM22α KO mice were more prone to heart failure after TAC.

**Conclusions:**

Our findings identified that SM22α may be required for the architecture and function of caveolae and T-tubules in cardiomyocytes.

## Introduction

Smooth muscle 22-alpha, or transgelin, is an actin-binding protein that is abundantly expressed in adult vascular smooth muscle cells (VSMCs) [1–4]. SM22α plays critical roles in mediating polymerization of both cortical and sarcomeric actin filaments and stretch sensitivity of cytoskeleton, which is important for maintaining the differentiated phenotype of VSMCs [1, 5–7]. Moreover, SM22α, as a scaffolding protein, mediates protein-protein interactions, and modulates the signaling activity in VSMCs [8–10]. The expression level of SM22α in VSMCs is sensitive to changes in blood pressure, as hypertension increases and mechanical unloading reduces its expression level [11]. Multiple lines of evidence indicate that SM22α may exist in cardiomyocytes [12–14]. However, the existence and physiological role of cardiac SM22α remain to be defined.

Specialized membrane structures, caveolae [15–17] and T-tubules [18], are emerging as essential components in mechanotransduction in cardiomyocytes. These structures allow the cardiac action potential to propagate into the interior of myocytes, initiating the process of excitation-contraction coupling (ECC). Both caveolae and T-tubules are shaped and maintained mainly by membrane scaffolding proteins such as cortical actins, as well as surrounding extracellular matrix [19]. A recent study suggested that caveolae function as a membrane reservoir to buffer membrane tension and to accommodate mechanical stresses [15]. It has been known that cortical actin cytoskeleton and actin-associated proteins are involved in shaping and maintaining the plasma membrane architecture in cardiomyocytes.

In the present study, we identified that the cardiomyocytes of SM22α KO mice displayed asynchronized SR Ca^2+^ release and abnormal contractile properties, accompanied with the disorganization of the plasma membrane architecture, making the heart more vulnerable to pressure overload. Our data suggested that SM22α may be required for the architecture and function of caveolae and T-tubules in cardiomyocytes.

## Materials and methods

An expanded Materials and Methods section is available in the S1 Protocol. SM22α KO mice (B6.129S6-Taglntm^2(cre)Yec^/J), purchased from the Jackson Laboratory. Pressure overload was produced by transverse aortic constriction (TAC). Mouse left ventricular myocytes were isolated by the Langendorff method, using retrograde perfusion through the aorta with enzyme-containing solutions. T-tubules of ventricular myocytes was visualized by Di-8-ANEPPS staining. For calcium imaging, isolated cardiomyocytes were stained with the Ca^2+^-sensitive dye Fluo-4-acetoxymethyl ester. Data are presented as mean ± SEM. One-way ANOVA and the Student t test was used for statistical analysis as appropriate. *P*<0.05 was considered statistically significant.

## Results

### Cardiac function is reduced in SM22α KO mice

**Fig 1:**
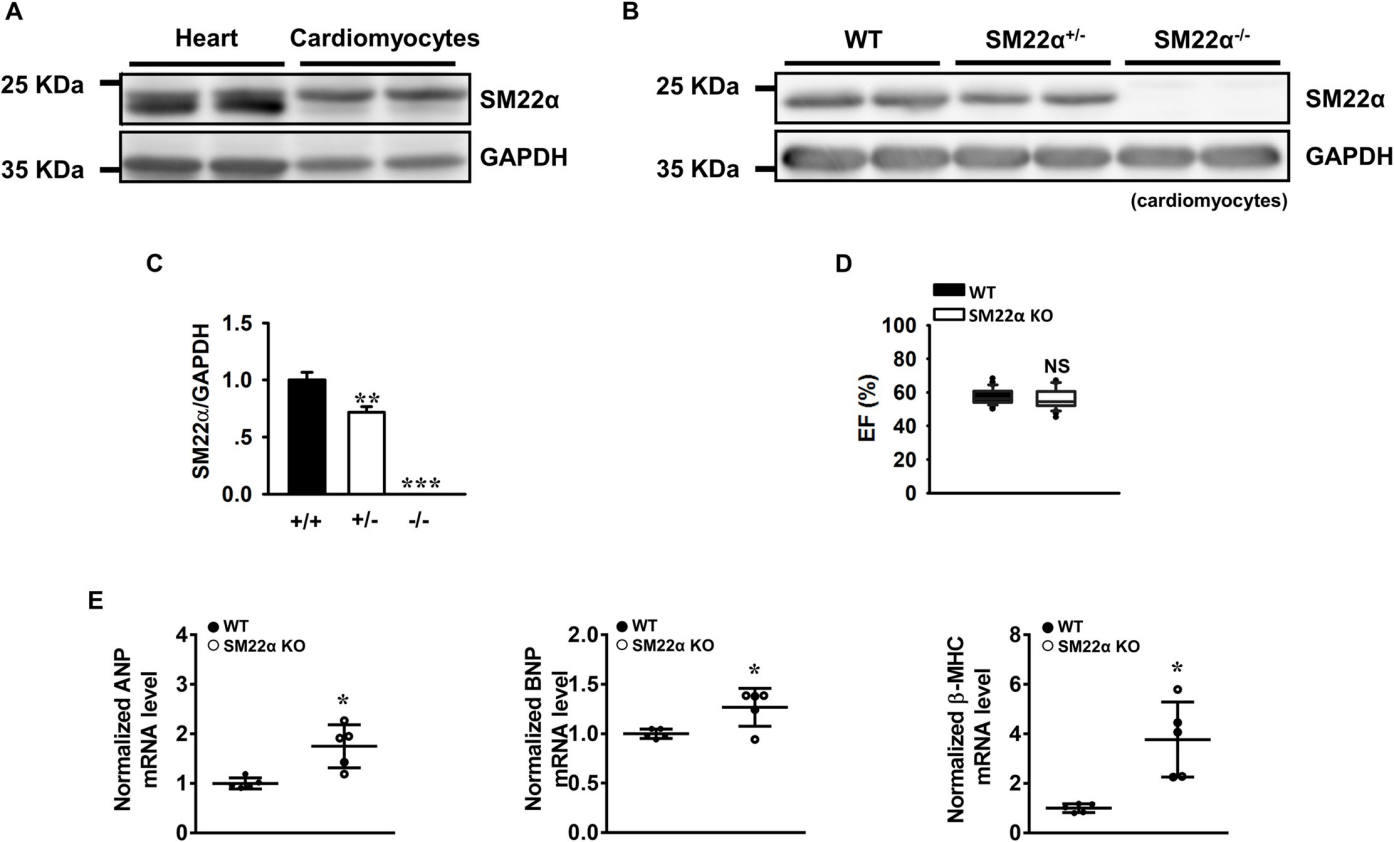
The cardiac function is reduced in SM22α KO mice. (A) and (B) Representative Western blot of the expression of SM22α protein in heart tissue and cardiomyocytes of WT, SM22α KO (SM22α^-/-^) and heterozygous (SM22α^+/-^) mice. (C) Bar graphs showing the expression of SM22α protein in WT (+/+), heterozygous (+/-) and KO (-/-) mice (n = 6 per group). (D) Echocardiographic assessment of ejection fraction (EF) (n = 40 per group). (E) RT-PCR analysis of cardiac fetal genes and hypertrophic markers ANP, BNP, β-MHC (n = 5 per group). All data are represented as mean ± SEM from 3 independent experiments. *P< 0.05, **P< 0.01, ***P< 0.001 vs. WT control.

It is still under debate whether SM22α exists in the heart growing into adulthood [2, 3, 13, 14, 20]. By using anti-SM22α antibody, here we showed that a band of approximately 22 kDa was detected in lysates of cardiac tissues and isolated cardiomyocytes from adult WT mice (Fig 1A), the expression level was the greatest in WT, less in heterozygous and was missing in the cardiomyocytes of SM22α KO mice (Fig 1B and 1C). Similarly, the expression of SM22α mRNA was validated by RT-PCR in the cardiomyocytes from adult WT mice, and increased in the cells of mice with heart failure (Fig 7A, S1 Fig). Next we examined the effect of SM22α disruption on cardiac function. Ejection fraction was slightly decreased in SM22α KO mice (55.93 ± 0.92%) compared with WT controls (57.36 ± 0.71%; Fig 1D), but the difference was statistically insignificant. SM22α KO mice also exhibit phenotypically normal vascular system under basal condition [5, 10, 21, 22]. However, the mRNA level of ANP, BNP and β-MHC was significantly increased in the myocardial tissues of SM22α KO mice (Fig 1E). Our finding validates that SM22α protein exists in the heart of adult mice, and the disruption of SM22α may induce a hypertrophic phenotype similar to that elicited by pressure overload.

### Disassembly and flattening of caveolae occur in the cardiomyocytes from SM22α KO mice

**Fig 2:**
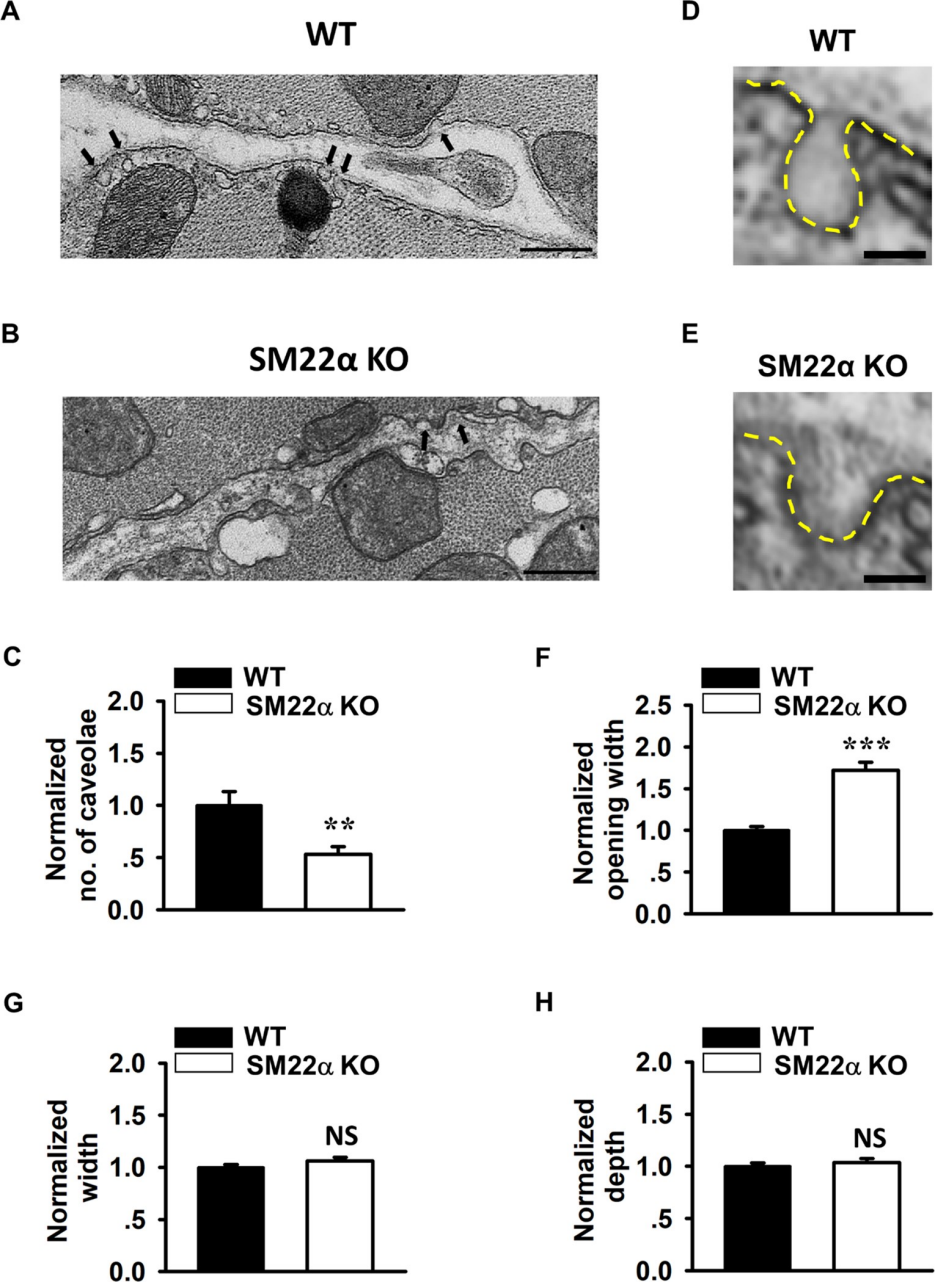
Disassembly and flattening of caveolae occur in the cardiomyocytes from SM22α KO mice. (A, B) Low-magnification electron micrographs of caveolae in the cardiomyocytes from WT (A) and SM22α KO (B) mice (arrowheads denote caveolae), Scale bars = 500 nm. (C) Bar graphs showing decreased caveolae density in the cardiomyocyte of SM22α KO mice (WT: 22 micrographs from 3 mice; KO: 27 micrographs from 3 mice). (D, E) Zoom-in images of representative caveolae. Yellow dashed-lines: highlighting the shape of representative caveolae. Scale bars = 100 nm. (F) Bar graphs showing increased opening width of caveolae in cardiomyocytes of SM22α KO mice (n = 67 caveolae from 3 WT mice and n = 77 caveolae from 3 KO mice). (G, H) Bar graphs showing no significant changes of caveolae maximal width (G) and depth (H) in cardiomyocytes of SM22α KO mice (n = 67 caveolae from 3 WT mice and n = 77 caveolae from 3 KO mice). Data are presented as the mean ± SEM. **P< 0.01, ***P< 0.001 vs. WT control.

Caveolae are membrane invaginations that play critical roles in cardiac mechano-protection during pressure overload [23]. Therefore, we next performed transmission electron microscopy to assess whether caveolae structure is altered in SM22α KO mice under basal condition. We showed that the density of caveolae on the plasma membrane was significantly reduced in the cardiomyocytes of SM22α KO mice (Fig 2A–2C). Importantly, the morphology of individual caveolae was strikingly altered under basal condition. As highlighted by the yellow dashed-lines in Fig 2D and 2E, the caveolae of WT cardiomyocytes displayed a classic flask-shape or omega-shape invagination that is characterized as a restricted neck region and an enlarged chamber (Fig 2D). However, the caveolae in SM22α KO cells were typically dome-shape invagination with enlarged opening width (Fig 2E). The opening width of SM22α KO caveolae was approximately 70% greater than the control (Fig 2F), with the maximum width and the depth of caveolae being comparable in KO and WT control animals (Fig 2G and 2H).

### The interaction between actin and Cav3 is decreased in SM22α KO cardiomyocyte

**Fig 3:**
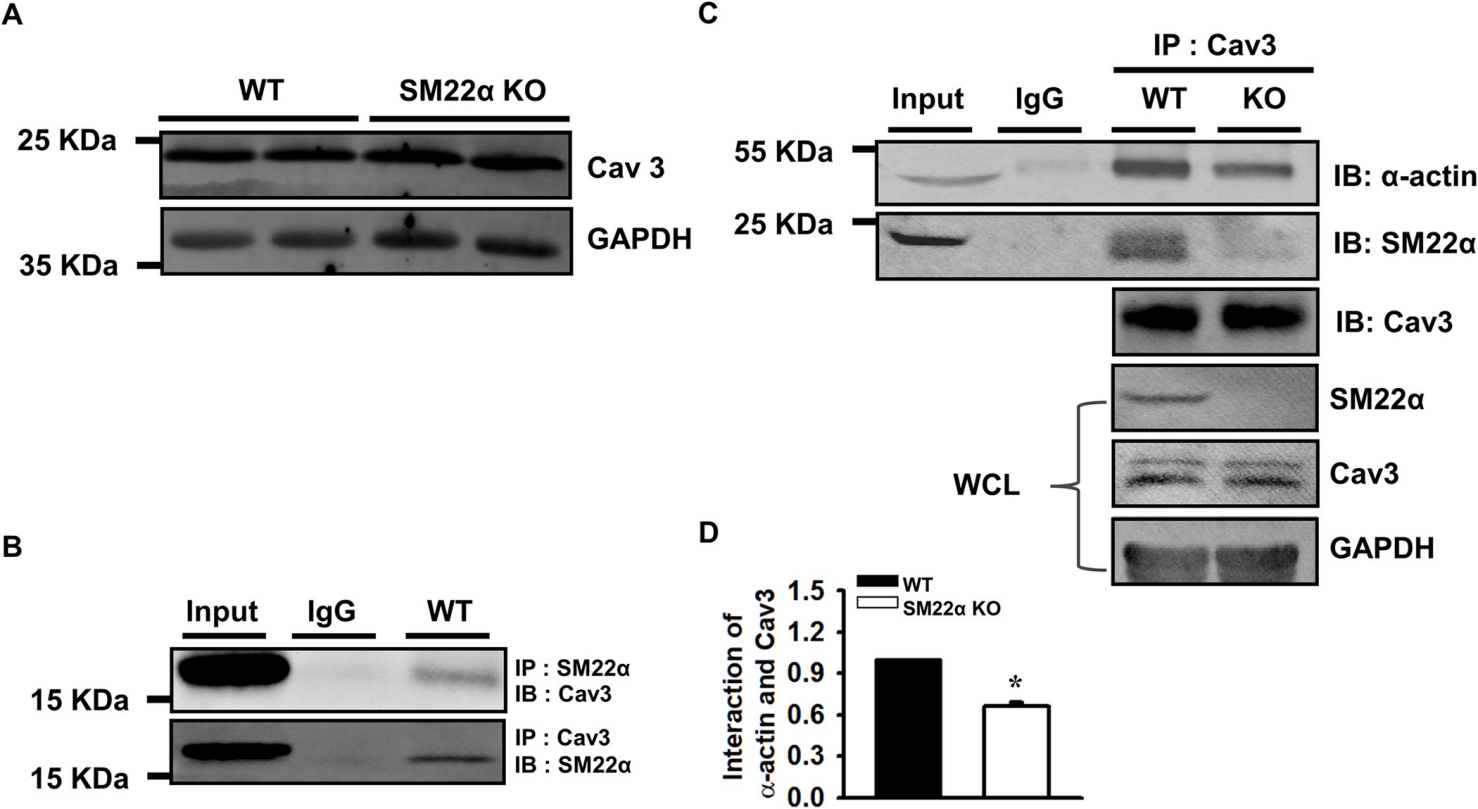
The interaction between actin and Cav3 is decreased in SM22α KO cardiomyocyte. (A) Western blot for the expression of Cav3. (B, C) Co-immunoprecipitation (co-IP) analysis for the interaction between SM22α, Cav3 and α-actin. Input lanes correspond to the original extracts used for the co-IP assays. IgG was used as negative control. (D) Bar graphs showed the quantification of α-actin-Cav3 interaction. WCL: whole cell lysate. Data are presented as the mean ± SEM from 3 independent experiments (n = 3). *P< 0.05 vs. WT control.

Cav3 is a major constituent of cardiac caveolae [24]. The expression level of Cav3 in SM22α KO heart is comparable to that in WT control (Fig 3A). Next we performed co-immunoprecipitate (Co-IP) to assess whether Cav3 interacts with SM22α or whether actin-Cav3 interaction is affected by SM22α deletion. We showed Cav3 was co-immunoprecipitated with anti-SM22α antibody and vice versa (Fig 3B). Additionally, we found that actin interacted with Cav3 (S2 Fig), and the interaction was reduced by 34% in SM22α KO cardiomyocytes (Fig 3C and 3D), implying that SM22α may confine caveolae to the plasma membrane through association with actin in the cells.

### T-tubules are disrupted in the cardiomyocytes of SM22α KO mice

**Fig 4:**
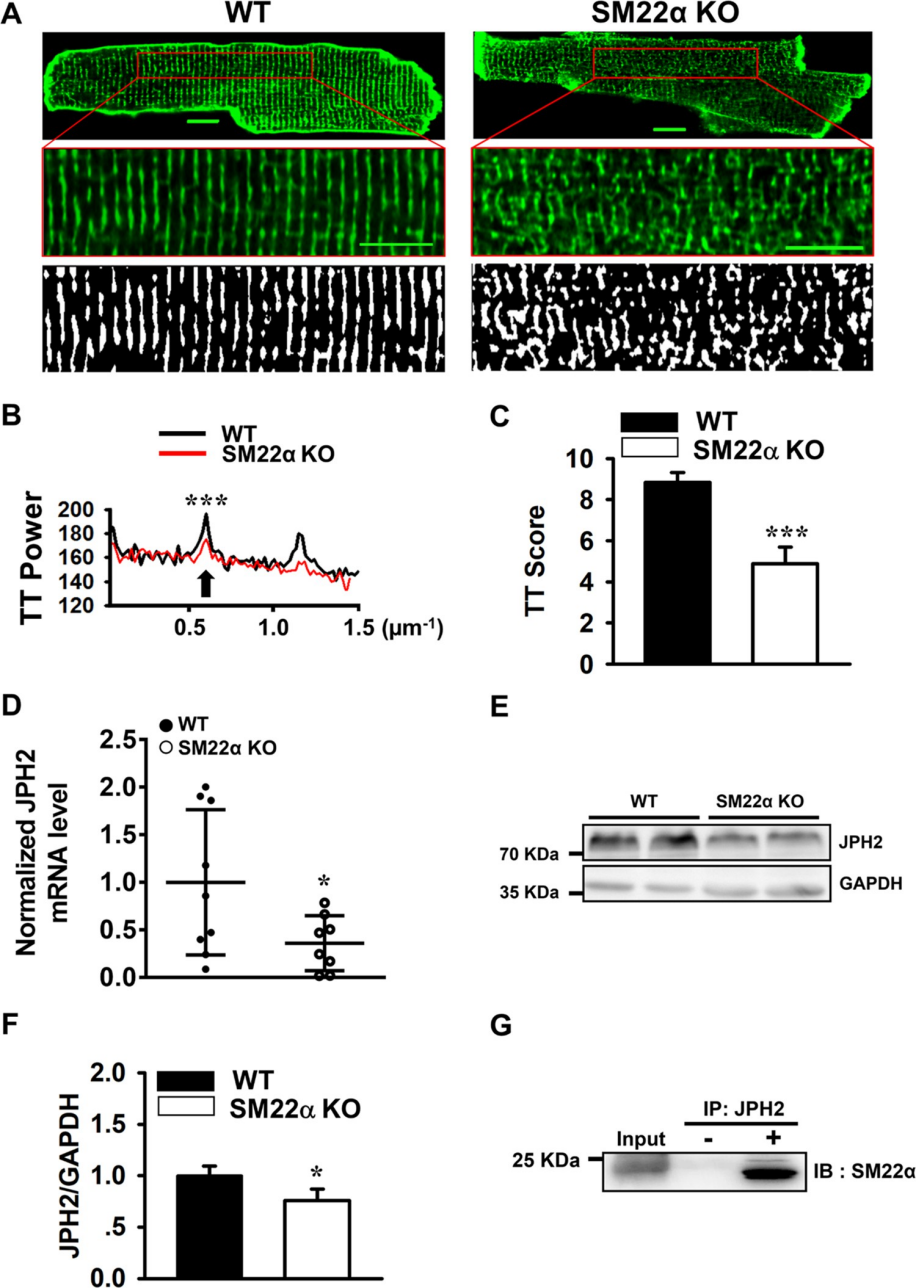
T-tubules are disrupted in SM22α KO cardiomyocytes. (A) Confocal fluorescence images of representative di-8-ANEPPS-stained single cardiomyocytes isolated from WT and SM22α KO mice (upper). Respective high-resolution views and of areas within the red rectangles (middle). Corresponding Fast Fourier transformed images of T-tubule images (bottom). Scale bar = 10 μm. (B) Representative power vs. spatial frequency along the x axis computed using Fourier analysis. (C) Bar graph showing TT score vs. WT control (n = 20 cells from 3 hearts in each group). (D) qRT-PCR analysis of JPH2 mRNA levels (WT: n = 9; KO: n = 8). (E) Western blot of JPH2 protein level in cardiomyocytes of WT and SM22α KO mice. (F) Bar graph showed the mean ± SEM from 3 independent experiments (n = 4 per group). (G) Co-IP analysis for the interaction of SM22α with JPH2. *P< 0.05, ***P< 0.001 vs. WT control.

The integrity of T-tubules is crucial in maintaining calcium homeostasis and normal contractile function in cardiomyocytes, and disrupted T-tubular system is a common feature in heart failure [25]. We stained plasma membrane with Di-8-ANEPPS and performed confocal microscopy to evaluate the integrity of T-tubules in SM22α KO mice. We showed that the well-organized T-tubule network observed in WT cardiomyocytes was disrupted in SM22α KO cells at baseline (Fig 4A), similar to the appearance of T-tubules in failing heart [26]. Quantification of T-tubule organization showed that the integrity of T-tubules was significantly impaired in SM22α KO cells, with reduced TT power and TT score (Fig 4B and 4C), suggesting that SM22α may be involved in the organization of T-tubules.

Transcriptomic analysis of the arteries of SM22α KO mice revealed a significant reduction in JPH2 mRNA level [27], which was validated by qRT-PCR in cardiac tissue. We found that the expression of JPH2 displayed an approximately 50% reduction in mRNA (Fig 4D) and 30% reduction in protein level (Fig 4E and 4F) in cardiomyocytes. Co-IP experiment further revealed that SM22α was immunoprecipitated with anti-JPH2 antibody (Fig 4G), suggesting that SM22α may promote the formation of well-organized T-tubule network via its interaction with JPH2.

### Disruption of SM22α is associated with abnormal SR Ca^2+^ handling in cardiomyocytes

**Fig 5:**
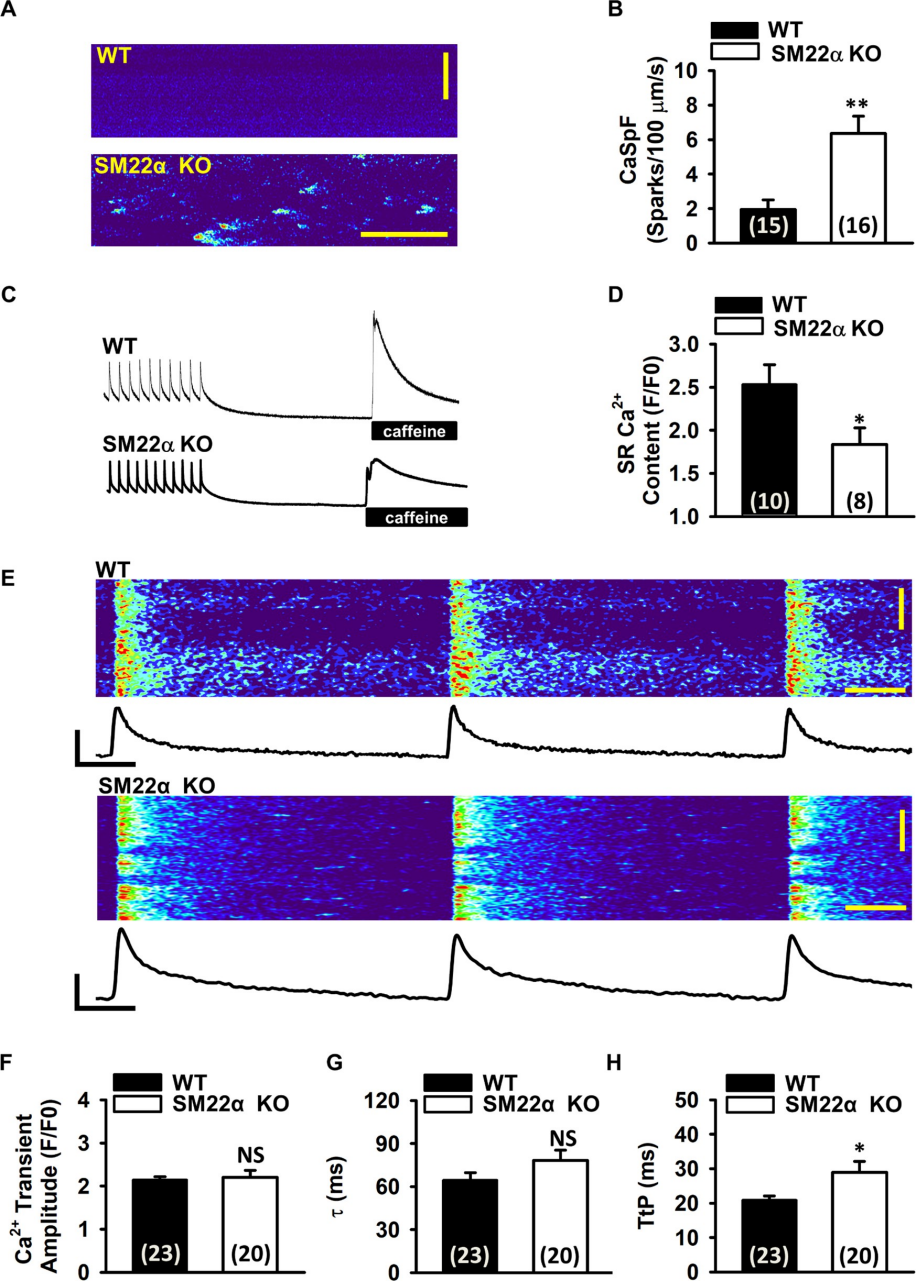
The disruption of SM22α is associated with abnormal SR Ca^2+^ handling in the cardiomyocytes. (A) Confocal microscopy line scans revealing increased number of Ca^2+^ sparks in SM22α KO mice. (B) Bar graph showing frequency of spontaneous Ca^2+^ sparks (n = 15 cells from 3 WT mice and 16 cells from 3 SM22α KO mice). (C) Representative tracings of Ca^2+^ transient amplitudes when paced at 1 Hz and total SR Ca^2+^ content as measured by the amplitude of caffeine-induced Ca^2+^ transient. (D) Bar graphs showing average SR Ca^2+^ content (n = 10 cells from 3 WT mice and 8 cells from 3 SM22α KO mice). (E) Representative confocal line scan images showing desynchronized Ca^2+^ transient in SM22α KO mice. (F) Bar graphs showing the average Ca^2+^ transient amplitude (n = 23 cells from 3 WT mice and 20 cells from 3 SM22α KO mice). (G) Bar graphs showing the Ca^2+^ decay constant (τ) (n = 23 cells from 3 WT mice and 20 cells from 3 SM22α KO mice). (H) Bar graphs showing the time to peak (TtP) (n = 23 cells from 3 WT mice and 20 cells from 3 SM22α KO mice). Data are presented as the mean ± SEM. *P< 0.05 vs. WT control.

T-tubule integrity is an important determinant of healthy Ca^2+^ release in cardiomyocytes [26]. To test whether T-tubule disruption correlated with defective Ca^2+^ release, we first used confocal line scan imaging to visualize Ca^2+^ sparks in isolated cardiomyocytes. SM22α KO cardiomyocytes displayed a significantly increased Ca^2+^ spark frequency (Fig 5A and 5B), accompanied with decreased SR Ca^2+^ content observed from a caffeine dump protocol (Fig 5C and 5D). Furthermore, we examined Ca^2+^ transients in both WT and SM22α KO cardiomyocytes with confocal microscopy in line-scan mode, and showed that there were abnormal Ca^2+^ transients in SM22α KO cells (Fig 5E). The amplitude of Ca^2+^ transients and Ca^2+^ decay constant (τ) were comparable between WT and SM22α KO cardiomyocytes (Fig 5F and 5G). However, the time to peak (TtP) was significantly prolonged in SM22α KO cells compared with WT control (Fig 5H), suggesting that disruption of T-tubules is associated with asynchronized SR Ca^2+^ release in SM22α KO cardiomyocytes.

### SM22α KO exhibits altered kinetics of cell shortening of cardiomyocytes, making the heart more vulnerable to pressure overload

**Fig 6:**
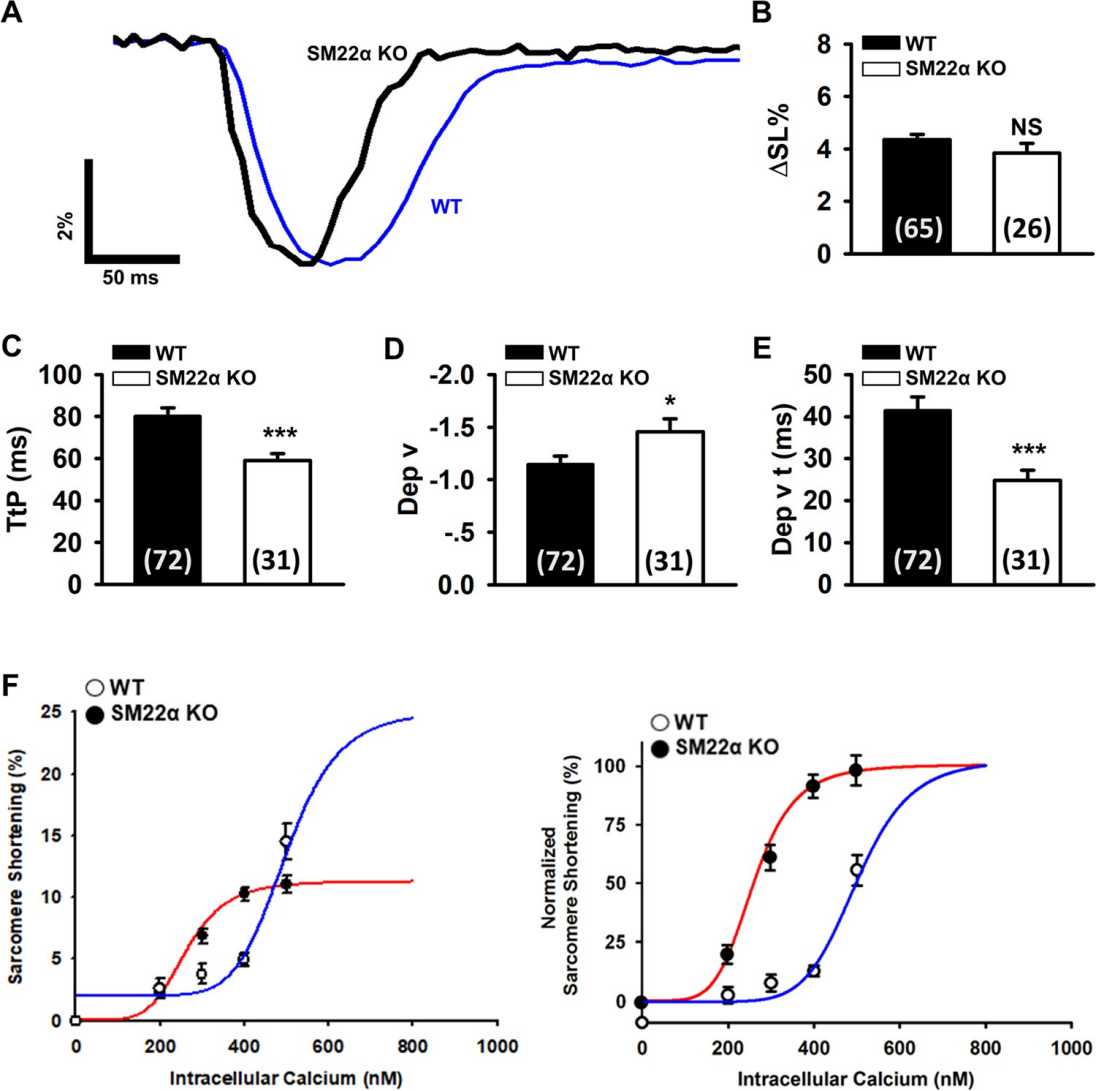
SM22α KO cardiomyocytes display altered kinetics of cell shortening. (A) Representative tracings showed altered kinetics of cell shortening in SM22α KO cardiomyocytes. (B) The amplitude of contraction of the cardiomyocytes. (C-E) Bar graphs showing decreased TtP (C), increased Dep v (D) and reduced Dep v t (E) in SM22α KO mice (n = 72 cells from 6 WT mice; n = 31 cells from 4 SM22α KO mice). *P< 0.05, ***P< 0.001 vs. WT control. (F) Perfused cells with 0, 200, 300, 400, 500 nmol/L Ca^2+^ buffer to record cell shortening in saponin permeabilized myocytes. Continuous lines showed Hill fits with Hill coefficients (n_H_) = -6.22 and concentration for 50% of maximal effect (EC_50_) = 515 nmol/L for WT cells, and n_H_ = -4.99, EC_50_ = 288 nmol/L for the KO cells (n = 9 or 10 from 3 mice in each group). Ca^2+^ sensitivity of myofilament was increased (right), but maximum response of myofilament was decreased in SM22α KO cells (left). Data are presented as the mean ± SEM. *P< 0.5, ***P< 0.001 vs. WT control.

The disruption of SM22α reduces the contractility of VSMCs [28]. Next we studied whether SM22α ablation alters kinetics of cell shortening in cardiomyocytes. We showed that the amplitude of cell shortening was reduced by approximately 12% in the cardiomyocytes of SM22α KO mice (3.843 ± 0.363%) compared with the WT controls (4.354 ± 0.201%), though it was statistically insignificant (Fig 6A and 6B). Additionally, the time to peak of cell shortening was significantly briefer and the maximum contraction velocity was significantly greater (Fig 6C–6E). Taken together, these data suggested that the kinetics of sarcomere shortening was altered in SM22α KO cardiomyocytes. To assess the effect of SM22α deletion on the Ca^2+^ sensitivity of myofilament, next we permeabilized the plasma membrane of cardiomyocytes with saponin and perfused the cells with the same set of intracellular buffers, then assessed the free Ca^2+^-sarcomere length relationship in WT and KO mice. We found that the Ca^2+^ sensitivity was increased, but the maximum response of sarcomere length to Ca^2+^ was reduced in comparison with WT controls (Fig 6F).

### The disruption of SM22α accelerates the development of heart failure induced by TAC

**Fig 7:**
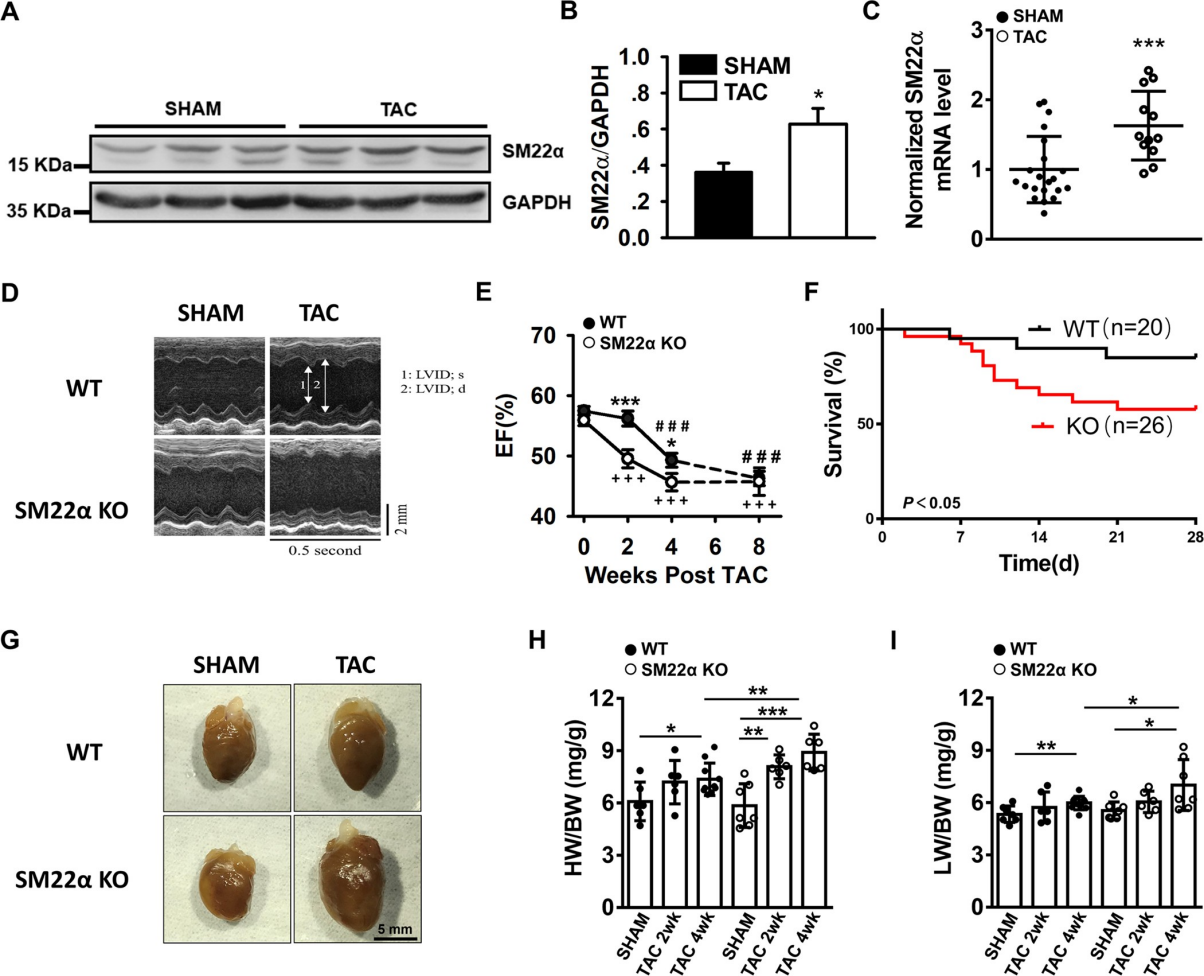
The disruption of SM22α accelerates the development of heart failure induced by TAC. (A) Western blot of SM22α in cardiomyocytes of WT mice sham and TAC hearts. (B) Bar graph showed increased level of SM22α protein post TAC (n = 5 in each group). (C) qRT-PCR analysis of SM22α mRNA (WT: n = 21; KO: n = 12). (D) Echocardiographic assessment of heart function using M-mode images of left ventricle. (E) Quantitative evaluation of systolic function in LV EF of 0, 2, 4 and 8 weeks post TAC (n = 35–40 per group). Data are presented as the mean ± SEM. *P< 0.5, ***P < 0.001 vs. WT control for the same week, ^###^*P*< 0.001 vs. WT baseline, ^+++^*P*< 0.001 vs. SM22α KO baseline. (F) Kaplan-Meier curve revealing increased mortality in SM22α KO mice compared with WT mice after TAC (WT: n = 20; KO: n = 26). (G) Whole mount of representative hearts from WT and SM22α KO sham or 4 weeks post TAC. (H, I) Heart weight to body weight ratio (HW/BW) (H) and lung weight to body weight ratio (LW/BW) (I) for WT and SM22α KO mice 2 and 4 weeks post TAC or sham surgery (n = 6–10 per group). Data are represented as mean ± SEM. *P< 0.05, **P< 0.01, ***P< 0.001.

To test our hypothesis, SM22α KO and WT mice were challenged with pressure overload induced by TAC. The expression of SM22α at protein and mRNA levels was increased in the cardiomyocytes of WT mice following TAC (Fig 7A–7C), implying that SM22α might be required for cardiac adaptive remodeling in response to pressure overload. Furthermore, the ejection fraction was significantly reduced in SM22α KO mice subjected to TAC for 2 weeks compared with WT control that exhibited similar reduction after TAC for 4 weeks (Fig 7D and 7E). Additionally, SM22α KO mice subjected to TAC had significantly reduced survival (Fig 7F), accompanied with increased heart weight and lung weight corrected to body weight, compared with WT mice (Fig 7G–7I). Taken together, our data demonstrated that the disruption of SM22α accelerates the development of heart failure induced by pressure overload.

## Discussion

Although SM22α is well recognized as a marker of VSMCs, it is still under debate whether it exists in the heart growing into adulthood [2, 3, 13, 14, 20]. We showed that SM22α is present in the heart tissues of adult mice, and is up-regulated upon pressure overload. To circumvent the potential contamination of SM22α from other tissues in the heart (such as coronary vessels), we enzymatically isolated and purified cardiomyocytes from the left ventricle, and indeed we detected SM22α protein and mRNA in the lysates from purified cardiomyocytes which increased the expression of SM22α in response to TAC. Our finding is consistent with multiple lines of evidence from other groups. Crossing SM22α-Cre mice with Nrp-1^flox/flox^ mice led to knockout of NRP-1 in both SMCs and cardiomyocytes [12]. Cre-mediated β-galactosidase expression was observed in SMCs and cardiac myocytes of heterozygous SM22α-Cre-R26R mice [13]. Additionally, eGFP SM22α-CreKI mouse exhibits cre recombinase activity in the cardiac myocytes of adults [14]. Taken together, here we provided evidence indicating the expression of SM22α in cardiomyocytes.

Caveolae have emerged as vital plasma membrane sensors that can respond to plasma membrane stresses [15, 29, 30]. Caveolae physically associate and functionally interplay with the actin cytoskeleton, particularly the stress fibers to adapt to the changing environment. We showed that the caveolae structure was altered in the cardiomyocytes of SM22α KO mice, which exhibited reduced density and the flattening morphology. The abnormal morphological alterations of the caveolae were accompanied with reduced interaction between actin and Cav3 in SM22α KO cells. Furthermore, we found that SM22α interacted with both actin and Cav3 in the WT cells, in accordance with a characteristic omega (Ω) shape, suggesting that SM22α may be required for the interaction between caveolae and actin cytoskeleton, and the disruption of SM22α may impair caveolae function in buffering mechanical stress at the plasma membrane via disassembly of caveolae. It has been known that caveolae associate with actin filaments, and the actin cytoskeleton is also shown by the flattening of caveolae in response to excessive actin polymerization [31]. This marked change of the caveolae may have a potentially significant impact on the ability of cardiomyocytes to handle increased stress following TAC. SM22α decorates the contractile filament bundles and promotes stress fiber formation in VSMCs exhibiting differentiated phenotypes via its interaction with SM α-actin [9, 32]. The down-regulation of SM22α was accompanied by decrease in stress fibers and increase in cortical actin rearrangement in synthetic VSMCs [33, 34]. The tension in the plasma membrane determines the curvature of caveolae because they flatten at high tension and invaginate at low tension, thus providing a tension-buffering system [35]. There was flattening of caveolae in SM22α KO cardiomyocytes, suggesting that SM22α may be a linker protein that can regulate the physical association of caveolae with stress fibers and bridge caveolae and stress fibers. It has been reported that Filamin A, an actin cross linker, anchors caveolae to stress fiber [36, 37]. The caveolae that are anchored to stress fibers remain static that is of omega-like invagination morphology observed from WT cardiomyocytes. Caveolae flattening and disassembly, together with increased expressions of *ANP*, *BNP* and *β-MHC*, may also represent an increased biomechanical stress in the cardiomyocytes of SM22α KO mice due to pressure overload. The association of SM22α with caveolae might fulfill a particular function that differentiates it from stress fibers.

T-tubule is a key structure in cardiac ECC [38]. Their functional junctions with the sarcoplasmic reticulum, or dyad, is critical to intracellular Ca^2+^ release. Recent data have indicated that the dyadic anchoring protein JPH2 is a key regulator of dyadic formation and maintenance. A central role of the T-tubule is to relay the electrical depolarization signal throughout the depth of the cardiomyocyte, thus it is critical to the synchronization of cell-wide Ca^2+^ release. T-tubule remodeling can have major impact on Ca^2+^ handling, and is a key mechanism of cardiac dysfunction. In the current study, we showed that the integrity of T-tubules was disrupted in SM22α KO cardiomyocytes at baseline, which displayed the disorganization of the T-tubule network similar to that of failing heart [39]. Caveolae are invaginations of the plasma membrane with a diameter of 60–80 nm and a characteristic omega (Ω) shape. Caveolae are abundantly present in mechanically stressed cells including cardiomyocytes [29, 40]. T-Tubules, on the other hand, are deeper invaginations of the surface membrane that occur at each Z-line [41]. We speculated that SM22α, as a scaffolding protein, may mediate the association of JPH2 with T-tubule via its interaction with JPH2, as it is able to regulate protein interactions either in an actin-dependent or actin-independent manner [8, 10, 42]. The mechanism underlying SM22α regulating the structural organization of T-tubule and its association with JPH2 expression remains to be identified. T-tubule remodeling is correlated with congestive heart failure [43, 44]. Disrupted T-tubules will lead to asynchronized SR Ca^2+^ release following depolarization. We found the asynchronized SR Ca^2+^ release and a significant higher Ca^2+^ spark frequency, accompanied with reduced SR Ca^2+^ content in SM22α KO cardiomyocytes. In addition, we also showed that most kinetic properties of cell shortening were significantly altered in SM22α KO cells, such as time to peak and maximal contraction velocity, although the amplitude of contraction is as same as controls. Furthermore, the maximum response of sarcomere length to Ca^2+^ was reduced and the Ca^2+^ sensitivity of myofilaments was increased, suggesting that the cardiomyocytes losing SM22α are tendency to overload, which is consistent with a subtle loss of cardiac function in SM22α KO mice. The ejection fraction of SM22α KO mice was already decreased only two weeks following TAC, whereas WT mice were still normal at this time point.

## Conclusion

SM22α in cardiomyocytes may act as a key element in the regulation of biomechanical stress, SR Ca^2+^ release and kinetic properties. SM22α KO mice have asynchronized SR Ca^2+^ release and abnormal contractile properties in cardiomyocytes, accompanied with altered membrane structure, making the heart more vulnerable to increased workload. Our findings suggest that SM22α may be required for the architecture and function of caveolae and T-tubules in cardiomyocytes. However, as our work does not provide direct causal evidence linking SM22α to the specialized membrane structure and cardiac functions, further *in vitro* and *in vivo* study of the effects of specific knockout/rescuing SM22α expression in cardiomyocytes on the reorganization and functions of caveolae and T-tubules are warranted.

## Supporting information

S1 Fig The mRNA level of SM22α in cardiomyocytes of WT mice and SM22α KO mice. RT-PCR analysis of SM22α in cardiomyocytes of WT mice and SM22α KO mice (n = 4 in each group). **P < 0.01.
(DOCX)

S2 Fig Co-immunoprecipitation (co-IP) analysis for the interaction between Cav3 and α-actin. The anti-Cav3 and anti-α-actin antibodies were used for immunoprecipitation (IP) or Western blot (IB). Input lanes correspond to the original heart extracts used for the co-IP assays. IgG was used as negative control.
(DOCX)

S1 Protocol Expanded materials and methods. (DOCX)

S1 Dataset (XLSX)

S1 Raw images (PDF)

## References

[1] Shapland C, Hsuan JJ, Totty NF, Lawson D. Purification and properties of transgelin: a transformation and shape change sensitive actin-gelling protein. J Cell Biol. 1993;121(5):1065–1073. doi: 10.1083/jcb.121.5.1065 8501116 PMC2119678

[2] Li L, Liu Z, Mercer B, Overbeek P, Olson EN. Evidence for serum response factor-mediated regulatory networks governing SM22alpha transcription in smooth skeletal and cardiac muscle cells. 1997 J;187(2):311–321. doi: 10.1006/dbio.1997.8621 9242426

[3] Lees-Miller JP, Heeley DH, Smillie LB. An abundant and novel protein of 22 kDa (SM22) is widely distributed in smooth muscles. Purification from bovine aorta. Biochem J. 1987;244(3):705–709. doi: 10.1042/bj2440705 3446186 PMC1148053

[4] Lees-Miller JP, Heeley DH, Smillie LB, Kay CM. Isolation and characterization of an abundant and novel 22-kDa protein (SM22) from chicken gizzard smooth muscle. J Biol Chem. 1987;262(7):2988–2993.. 3818630

[5] Zeidan A, Swärd K, Nordström I, Ekblad E, Zhang JCL, Parmacek MS, et al. Ablation of SM22alpha decreases contractility and actin contents of mouse vascular smooth muscle. FEBS Lett. 2004;562(1–3):141–146. doi: 10.1016/S0014-5793(04)00220-0 15044015

[6] Zeidan A, Purdham DM, Rajapurohitam V, Javadov S, Chakrabarti S, Karmazyn M. Leptin Induces Vascular Smooth Muscle Cell Hypertrophy through Angiotensin II-and Endothelin-1-Dependent Mechanisms and Mediates Stretch-Induced Hypertrophy, J Pharmacol Exp Ther.2005;315(3):1075–1084. doi: 10.1124/jpet.105.091561 16144973

[7] Rattan S, Ali M. Role of SM22 in the differential regulation of phasic versus tonic smooth muscle. Am J Physiol Gastrointest. Liver Physiol.2015;308(7):G605–G612. doi: 10.1152/ajpgi.00360.2014 25617350 PMC4385893

[8] Shu YN, Dong LH, Li H, Pei QQ, Miao SB, Zhang F, et al. CKII-SIRT1-SM22α loop evokes a self-limited inflammatory response in vascular smooth muscle cells. Cardiovasc Res. 2017;113(10):1198–1207. doi: 10.1093/cvr/cvx048 28419207

[9] Lv P, Zhang F, Yin YJ, Wang YC, Gao M, Xie XL, et al. SM22α inhibits lamellipodium formation and migration via Ras-Arp2/3 signaling in synthetic VSMCs. Am J Physiol Cell Physiol. 2016;311(5):C758–C767. doi: 10.1152/ajpcell.00033.2016 27629412

[10] Shu YN, Zhang F, Bi W, Dong LH, Zhang DD, Chen R, et al.SM22α inhibits vascular inflammation via stabilization of IκBα in vascular smooth muscle cells. J Mol CellCardiol.2015;84:191–199. doi: 10.1016/j.yjmcc.2015.04.020 25937534

[11] Liu R, Hossain MM, Chen X, Jin JP. Mechanoregulation of SM22α/Transgelin., Biochemistry.2017;56(41):5526–5538. doi: 10.1021/acs.biochem.7b00794 28898058

[12] Wang Y, Cao Y, Yamada S, Thirunavukkarasu M, Nin V, Joshi M, et al. Cardiomyopathy and Worsened Ischemic Heart Failure in SM22-α Cre-Mediated Neuropilin-1 Null Mice: Dysregulation of PGC1α and Mitochondrial Homeostasis., Arterioscler Thromb Vasc Biol. 2015;35(6):1401–1412. doi: 10.1161/ATVBAHA.115.305566 25882068 PMC4441604

[13] Lepore JJ, Cheng L, Min M, Mericko PA, Morrisey EE, Parmacek MS. High-efficiency somatic mutagenesis in smooth muscle cells and cardiac myocytes in SM22alpha-Cre transgenic mice. Genesis. 2005;41(4):179–184. doi: 10.1002/gene.20112 15789423

[14] Zhang J, Zhong W, Cui T, Yang M, Hu X, Xu K, et al. Generation of an adult smooth muscle cell-targeted Cre recombinase mouse model. Arterioscler Thromb Vasc Biol. 2006;26(3):e23–e24. doi: 10.1161/01.ATV.0000202661.61837.93 16484601 PMC4207359

[15] Sinha B, Köster D, Ruez R, Gonnord P, Bastiani M, Abankwa D, et al. Cells Respond to Mechanical Stress by Rapid Disassembly of Caveolae. Cell. 2011;144(3):402–413. doi: 10.1016/j.cell.2010.12.031 21295700 PMC3042189

[16] Insel PA, Patel HH. Membrane rafts and caveolae in cardiovascular signaling, Curr Opin Nephrol Hypertens. 2009;18(1):50–56. doi: 10.1097/MNH.0b013e3283186f82 19077689 PMC2757134

[17] Gratton JP, Bernatchez P, Sessa WC. Caveolae and caveolins in the cardiovascular system. CircRes. 2004;94(11):1408–1417. doi: 10.1161/01.RES.0000129178.56294.17 15192036

[18] Crossman DJ, Shen X, Jüllig M, Munro M, Hou Y, Middleditch M, et al. Increased collagen within the transverse tubules in human heart failure. Cardiovasc Res. 2017;113(8):879–891. doi: 10.1093/cvr/cvx055 28444133

[19] Hong T, Shaw RM. Cardiac T-Tubule Microanatomy and Function. Physiol Rev. 2017;97(1):227–252. doi: 10.1152/physrev.00037.2015 27881552 PMC6151489

[20] Yang M, Jiang H, Li L. Sm22α transcription occurs at the early onset of the cardiovascular system and the intron 1 is dispensable for its transcription in smooth muscle cells during mouse development. Int J Physiol. 2010;2(1):12–19. Epub 2009 Nov 22. 20428474 PMC2860299

[21] Zhang JC, Kim S, Helmke BP, Yu WW, Du KL, Lu MM, et al. Analysis of SM22alpha-deficient mice reveals unanticipated insights into smooth muscle cell differentiation and function. Mol Cel Biol.2001;21(4):1336–1344. doi: 10.1128/MCB.2001.21.4.1336-1344.2001 11158319 PMC99586

[22] Feil S, Hofmann F, Feil R.SM22α Modulates Vascular Smooth Muscle Cell Phenotype during Atherogenesis. Circ Res. 2004;94(7):863–865. doi: 10.1161/01.RES.0000126417.38728.F6 15044321

[23] Wei EQ, Sinden DS, Mao L, Zhang H, Wang C, Pitt GS. Inducible Fgf13 ablation enhances caveolae-mediated cardioprotection during cardiac pressure overload. Proc Natl Acad Sci U S A. 2017;114(20):E4010–E4019. doi: 10.1073/pnas.1616393114 28461495 PMC5441822

[24] Song KS, Scherer PE, Tang Z, Okamoto T, Li S, Chafel M, et al. Expression of caveolin-3 in skeletal, cardiac, and smooth muscle cells. Caveolin-3 is a component of the sarcolemma and co-fractionates with dystrophin and dystrophin-associated glycoproteins. J BiolChem.1996;271(25):15160–15165. doi: 10.1074/jbc.271.25.15160 8663016

[25] GuoY Vandusen NJ, Zhang L, Gu W, Sethi I, Guatimosim S, et al. Analysis of Cardiac Myocyte Maturation Using CASAAV, a Platform for Rapid Dissection of Cardiac Myocyte Gene Function in Vivo. Circ Res. 2017;120(12):1874–1888. doi: 10.1161/CIRCRESAHA.116.310283 28356340 PMC5466492

[26] Manfra O, Frisk M, Louch WE. Regulation of Cardiomyocyte T-Tubular Structure: Opportunities for Therapy. Curr Heart Fail Rep. 2017;14(3):167–178. doi: 10.1007/s11897-017-0329-9 28447290 PMC5423965

[27] Chen R, Zhang F, Song L, Shu YN, Lin Y, Dong LH, et al. Transcriptome profiling reveals that the SM22α-regulated molecular pathways contribute to vascular pathology. J Mol Cell Cardiol. 2014;72:263–272. doi: 10.1016/j.yjmcc.2014.04.003 24735829

[28] Xie XL, Nie X, Wu J, Zhang F, Zhao L, Lin YL, et al. Smooth muscle 22α facilitates angiotensin II-induced signaling and vascular contractio. J Mol Med (Berl). 2015;93(5):547–558. doi: 10.1007/s00109-014-1240-4 25515236

[29] Parton RG, Del Pozo MA. Caveolae as plasma membrane sensors, protectors and organizers.Nat Rev Mol Cell Biol. 2013;14(2):98–112. doi: 10.1038/nrm3512 23340574

[30] Cheng JP, Mendoza-Topaz C, Howard G, Chadwick J, Shvets E, Cowburn AS, et al. Caveolae protect endothelial cells from membrane rupture during increased cardiac output. J Cell Biol. 2015;211(1):53–61. doi: 10.1083/jcb.201504042 26459598 PMC4602045

[31] Parton RG. Caveolae: Structure, Function, and Relationship to Disease. Annu Rev Cell Dev Biol. 2018;34(1):111–136. doi: 10.1146/annurev-cellbio-100617-062737 30296391

[32] Han M, Dong L, Zheng B, Shi J, Wen J, Cheng Y. Smooth muscle 22 alpha maintains the differentiated phenotype of vascular smooth muscle cells by inducing filamentous actin bundling. Life Sci. 2009;84(13–14):394–401. doi: 10.1016/j.lfs.2008.11.017 19073196

[33] Lv P, Miao SB, Shu YN, Dong LH, Liu G, Xie XL, et al. Phosphorylation of smooth muscle 22α facilitates angiotensin II-induced ROS production via activation of the PKCδ-P47phox axis through release of PKCδ and actin dynamics and is associated with hypertrophy and hyperplasia of vascular smooth muscle cells in vitro and in vivo. Circ Res. 2012;111(6):697–707. doi: 10.1161/CIRCRESAHA.112.272013 22798525

[34] Zhao LL, Zhang F, Chen P, Xie XL, Dou YQ, Lin, YL, et al. Insulin-independent GLUT4 translocation in proliferative vascular smooth muscle cells involves SM22α. J Mol Med (Berl). 2017;95(2):181–192. doi: 10.1007/s00109-016-1468-2 27631639

[35] Echarri A, Del Pozo MA. Caveolae-mechanosensitive membrane invaginations linked to actin filaments. J Cell Sci. 2015;128(15):2747–2758. doi: 10.1242/jcs.153940 26159735

[36] Muriel O, Echarri A, Hellriegel C, Pavon DM, Beccari L, Del Pozo MA. Phosphorylated filamin A regulates actin-linked caveolae dynamics. J Cell Sci. 2011;124(Pt 16):2763–2776. doi: 10.1242/jcs.080804 21807941

[37] Stossel TP, Condeelis J, Cooley L, Hartwig JH, Noegel A, Schleicher M, et al. Filamins as integrators of cell mechanics and signalling. Nat Rev Mol Cell Biol. 2001;2(2):138–145. doi: 10.1038/35052082 11252955

[38] Kong CHT, Rog-Zielinska EA, Orchard CH, Kohl P, Cannell MB. Sub-microscopic analysis of t-tubule geometry in living cardiac ventricular myocytes using a shape-based analysis method. JMol Cell Cardiol. 2017;108:1–7. doi: 10.1016/j.yjmcc.2017.05.003 28483597 PMC5529290

[39] Van Oort RJ, Garbino A, Wang W, Dixit SS, Landstrom AP, Gaur N, et al. Disrupted junctional membrane complexes and hyperactive ryanodine receptors after acute junctophilin knockdown in mice. Circulation. 2011;123(9):979–988. doi: 10.1161/CIRCULATIONAHA.110.006437 21339484 PMC3056402

[40] Lyon RC, Zanella F, Omens JH, Sheikh F. Mechanotransduction in cardiac hypertrophy and failure. Circ Res. 2015;116(8):1462–1476. doi: 10.1161/CIRCRESAHA.116.304937 25858069 PMC4394185

[41] Soeller C, Cannell MB. Examination of the transverse tubular system in living cardiac rat myocytes by 2-photon microscopy and digital image-processing techniques. Circ Res. 1999;84(3):266–275. doi: 10.1161/01.res.84.3.266 10024300

[42] Miao SB, Xie XL, Yin YJ, Zhao LL, Zhang F, Shu YN, et al. Accumulation of Smooth Muscle 22α Protein Accelerates Senescence of Vascular Smooth Muscle Cells via Stabilization of p53 In Vitro and In Vivo. Arterioscler Thromb Vasc Biol. 2017;37(10):1849–1859. doi: 10.1161/ATVBAHA.117.309378 28798142

[43] Wang SQ, Song L, Lakatta EG, Cheng H. Ca2+ signalling between single L-type Ca2+ channels and ryanodine receptors in heart cells. Nature. 2001;410(6828):592–596. doi: 10.1038/35069083 11279498

[44] Brette F, Orchard C. T-tubule function in mammalian cardiac myocytes. Circ Res. 2003;92(11):1182–1192. doi: 10.1161/01.RES.0000074908.17214.FD 12805236

