# Supplementary figures and images for: Deletion of SM22α disrupts the structure and function of caveolae and T-tubules in cardiomyocytes, contributing to heart failure
Source: PLoS One. 2022 Jul 18;17(7):e0271578. doi: 10.1371/journal.pone.0271578 (PMC9292107; doi:10.1371/journal.pone.0271578)

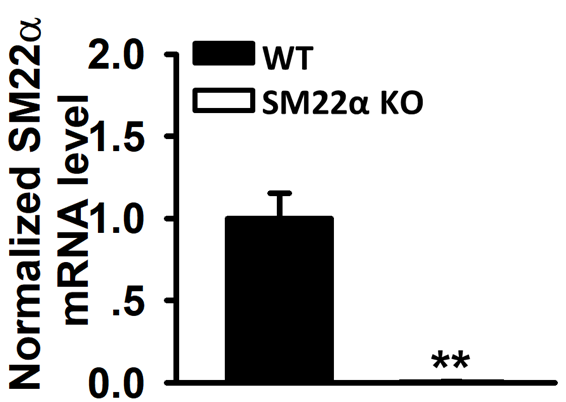


S1 Fig

Supplement: S1 Fig — RT-PCR analysis of SM22α in cardiomyocytes of WT mice and SM22α KO mice (n = 4 in each group). **P < 0.01. (DOCX) [file pone.0271578.s001.docx]

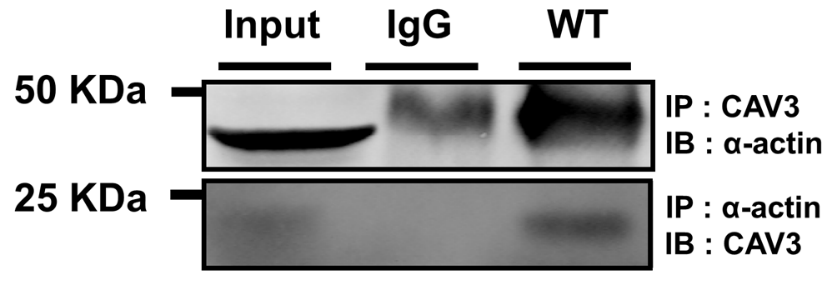


S2 Fig

Supplement: S2 Fig — The anti-Cav3 and anti-α-actin antibodies were used for immunoprecipitation (IP) or Western blot (IB). Input lanes correspond to the original heart extracts used for the co-IP assays. IgG was used as negative control. (DOCX) [file pone.0271578.s002.docx]
