# Supplementary material for: Deletion of SM22α disrupts the structure and function of caveolae and T-tubules in cardiomyocytes, contributing to heart failure
Source: PLoS One. 2022 Jul 18;17(7):e0271578. doi: 10.1371/journal.pone.0271578 (PMC9292107; doi:10.1371/journal.pone.0271578)
Supplement: S1 Protocol — (DOCX) [file pone.0271578.s003.docx]

**Expanded materials and methods**

**Animals and ethics statement**

All animal procedures conformed to the Guide for the Care and Use of Laboratory Animals published by the US National Institutes of Health (NIH Publication, 8th Edition, 2011) and this study was performed via a protocol approved by the Institutional Animal Care and Use Committee of Hebei Medical University, in accordance with the Guide for the Care and Use of Laboratory Animals. SM22α KO mice (B6.129S6-Taglntm^2(cre)Yec^/J) were purchased from the Jackson Laboratory. The mice were housed in the temperature- and humidity-controlled facility, with standard laboratory chow and *ad libitum* access to food and water [1].

**Transverse aortic constriction**

SM22α KO mice and littermate controls [wild-type (WT)] mice (12-20 week aged) were used. Pressure overload was produced by transverse aortic constriction (TAC) as described [2]. Mice were anesthetized in an induction chamber with 2% isoflurane mixed with 1.0 L/min 100% O_2_. The transverse aorta was mildly constricted with a 25-gauge needle using 6.0 silk sutures, after which the chest was closed and the mice were allowed to recover from anesthesia on a heating pad. In sham control mice, the entire procedure was identical except for the ligation of the aorta.

**Western blot analysis**

Lysates from left ventricular tissues were prepared with lysis buffer [(mmol/L): 150 NaCl, 50 Tris-HCl, pH 7.4, 5 EDTA, 5 EGTA, pH 8.0, 1 Na_3_VO_4_, 10 NaF, 0.5% Triton X-100, 0.5% Na-deoxycholate, 0.1% SDS and protease inhibitors (Sigma, P8340)] [3]. Equal amounts of protein (60~100 μg) were separated by 10% SDS-PAGE, and electrotransferred to a PVDF membrane. Membranes were blocked with 5% skimmed milk in Tris-buffered saline with 0.1% Tween 20 (TBST) for 2 hours at room temperature, and then incubated with specific primary antibodies [anti-SM22α (ab14102, Abcam, US), anti-Cav3 (ab2912, Abcam, US), anti-actin (ab179467, Abcam, US), anti-JPH2 (40-5300, Thermo Fisher)] at 4°C overnight. After that the membranes were incubated with the horseradish peroxidase (HRP) - conjugated secondary antibody (1:20000, Santa Cruz Biotechnology) for 1 hour. The blots were evaluated with the ECL (enhanced chemiluminescence) detection system. Alternatively, membranes were incubated with IRDye800® conjugated secondary antibody (1:20000, Rockland) for 1 hour, following scanning with the Odyssey Infrared Imaging System (LI-COR Biosciences). Each detected band was quantified with the ImageJ software (NIH). GAPDH was used as an internal control. These experiments were replicated at least three times.

**Isolation of cardiomyocytes.**

Mouse left ventricular myocytes were isolated using retrograde perfusion through the aorta with enzyme-containing solutions by the Langendorff method, as described [4,5]. Briefly, the heart was quickly removed and the blood was washed out with 0 Ca^2+^ Tyrode solution [(mmol/L): 137 NaCl, 5.4 KCl, 1 MgCl_2_, 5 HEPES, 10 glucose, 2.7 NaOH, pH 7.4]. The heart was perfused through the aorta on a Langendorff apparatus with 0 Ca^2+^ Tyrode for 3 to 5 minutes at 37 °C, followed by 0 Ca^2+^ Tyrode containing 20 μg/mL Liberase (Roche, Indianapolis, IN) for 20 to 30 minutes at 37 °C. After digestion, the heart was removed from the Langendorff apparatus and perfused with 5 mL KB solution [(mmol/L): 90 KCl, 30 K_2_HPO4, 5 MgSO_4_, 5 Na-Pyruvic acid, 5 Na-β-OH-Butyric acid, 5 creatine, 20 taurine, 10 glucose, 0.5 EGTA, 5 HEPES, pH 7.2]. Left ventricle was then minced in KB solution and gently agitated, then the suspension was filtered through a 200 µm polyethylene mesh. Cardiomyocytes were kept in KB solution at room temperature before use.

**Quantitative Real-Time RT-PCR (qRT-PCR)**

Total RNA from cardiomyocytes or left ventricular tissues were extracted using TRIzol Reagent (Invitrogen). The concentration and purity of RNA was measured by NanoDrop ND-2000 spectrophotometer (NanoDrop Technologies, USA), then the cDNA was synthesized using the reverse transcriptase (Invitrogen). Quantitative Real-time PCR was performed using SYBR Green RT-PCR Kit (Invitrogen) following manufacturer’s instructions. The relative mRNA expression was normalized to GAPDH.

Sequence-specific primers used were presented as following: SM22α (5’- CAACAAGGGTCCATCCTACGG-3’ and 5’- ATCTGGGCGGCCTACATCA-3’), GAPDH (5’- CAACGACCCCTTCATTGACC-3’ and 5’- ACACCAGTAGACTCCACGACA-3’), ANP (5’-CCTGATGGATTTCAAGAATTTGCTGGA-3’ and 5’-CTGCTTCCTCAGTCTGCTCACT-3’), BNP (5’-GCCAGTCTCCAGAGCAATTC-3’ and 5’-CCGATCCGGTCTATCTTGTG-3’), β-MHC (5’-GCCAACACCAACCTGTCCAAGTTC-3’ and 5’-TGCAAAGGCTCCAGGTCTGAGGGC-3’), JPH2 (5’-ACCTATCAAGGCCAATTCACCA-3’ and 5’-CCACCACTGCCATCCCGTA-3’), Cav3 (5’-GATGATGGCAGAAGAGCACA-3’ and 5’-GTGGACAACAGACGGTAGCA-3’)

**Transthoracic echocardiography**

Mice were anesthetized with 1% to 2% isoflurane mixed with 1.0 L/min 100% O_2_. Cardiac function was measured with M-mode echocardiograms by Vevo 770 (VisualSonics, Canada), as described [6].

**Electron microscopy**

The density and morphology of caveolae were imaged by electron microscopy as described [7,8]. Acutely dissected small section of left ventricles from WT or SM22α KO mice hearts were fixed in 2.5 % glutaraldehyde with 0.1 mol/L phosphate buffer (pH 7.4) overnight at 4 °C. Tissue were post fixed for 1 h in 1% osmium tetroxide in 0.1 mol/L phosphate buffer, dehydrated through acetone series, and embedded in resin and polymerized at 70 °C overnight. Ultrathin sections were cut from tissue blocks, counterstained with uranyl acetate and lead citrate, and examined using a model H-7500 transmission electron microscope (Hitachi, Matsumoto, Japan). The images of electron microscopy were imported into ImageJ (NIH) following acquisition, caveolae density and morphology were measured by two independent observers. Caveolae density was calculated from each image as the number of caveolae normalized to the length of membrane, then the number was normalized to WT.

**Co-immunoprecipitation assay**

Mouse left ventricular samples were prepared in a buffer composed (mmol/L): 20 Tris-HCl (pH 7.4), 150 NaCl, 1 Na_2_EDTA, 1 EGTA, 1% Triton, protease inhibitor (cOmplete™ Protease Inhibitor Cocktail, Roche), phosphatase inhibitor (PhosSTOP™, Roche). After centrifugation, the extracts were first precleared with 10 μL of protein G/A-agarose (Santa Cruz Biotechnology, CA). Then the supernatants were immunoprecipitated with specific antibodies with rocking at 4 ℃ overnight, followed by incubation with protein G/A-agarose for 2 hours at 4 ℃. After that, the immune complexes were collected by centrifugation at 4 ℃, and then washed for four times with ice-cold lysis buffer. The immunoprecipitated protein was further assessed by Western blot as described above. All blots are representative of three similar experiments.

**T-tubules imaging and analysis**

T-tubules of cardiomyocytes was visualized by incubating with 10 μmol/L Di-8-ANEPPS in 1.8 mmol/L Ca^2+^ Tyrode solution [(mmol/L): 137 NaCl, 5.4 KCl, 1 MgCl_2_, 5 HEPES, 10 glucose, 2.7 NaOH, pH 7.4) staining] for 10 min, as described [7]. Regions of interest were selected within a cell but outside of nucleus. Power spectrum was computed using Fast Fourier Transform (FFT). Spatial integrity of T-tubules (TT Power) was calculated as normalized power at spatial frequency of ~0.55 μm^-1^ (peak power at ~0.55 μm^-1^ normalized to average power at spatial frequency of 0.2 to 0.4 μm^-1^). We also defined a score to quantify the integrity of T-tubules, and quantitative analysis of spatial integrity of T-tubules was measured by two independent observers blinded to genotype.

**Ca^2+^ imaging**

Isolated cardiomyocytes were stained with the Ca^2+^-sensitive dye Fluo-4-acetoxymethyl ester (Fluo-4 AM, Invitrogen, CA) as described [7]. Cardiomyocytes were incubated with 2 μmol/L Fluo-4 AM in 1.8 mmol/L Ca^2+^ Tyrode solution for 30 minutes at room temperature. Cells were then washed with dye-free 1.8 mmol/L Ca^2+^ Tyrode solution for 15 minutes for de-esterification, followed by transferring to a chamber with a pair of parallel electrodes, Ca^2+^ imaging were recorded using a laser scanning confocal microscope (DMi 8, Leica, NY). After pacing at 1 Hz for at least 2 minutes, steady state Ca^2+^ transients were observed and recorded, then the pacing was stopped for about 45 seconds and spontaneous Ca^2+^ release events and Ca^2+^ sparks were counted. After pacing, 10 mmol/L caffeine was applied rapidly to estimate the steady state sarcoplasmic reticulum Ca^2+^ load.

**Cell shortening**

Contractile properties of mouse cardiomyocytes were assessed using a video-based myocyte sarcomere spacing acquisition system [IonOptix (Milton, MA)], as described [9]. Cardiomyocytes were placed in a chamber equipped with platinum electrodes on the stage of an inverted microscope, then perfused the cells with 1.8 mmol/L Ca^2+^ Tyrode solution. The stimulation frequency of the electric field was 1 Hz, and the changes in average sarcomere length were determined by fast Fourier transform (FFT) of the Z-line density trace to the frequency domain.

**Ca^2+^ sensitivity**

Isolated cardiomyocytes were permeabilized by exposure to the saponin β-escin (30 µmol/L) added to an intracellular solution contained (mmol/L): 125 KCl, 10 NaCl, 10 HEPES, and 1 EGTA, pH 7.2 at 22 °C with KOH. Next the cells were perfused with Ca^2+^-EGTA buffers contained (mmol/L): 125 KCl, 10 NaCl, 10 HEPES, and 1 EGTA, pH 7.2 at 22 °C with KOH with different [Ca^2+^]_i_ (200-500 nmol/L). [Ca^2+^]_i_ in these buffers was calculated as described previously [10]. Steady state sarcomere length was detected and the sarcomere length-[Ca^2+^]_i_ relationship was fitted with the equation as described [11]:

$$SL={SL}_{min}+\frac{\mathrm{SL}_{\max}-\mathrm{SL}_{\min}}{1+{(\frac{\left[ \mathrm{Ca}^{2+} \right]_{i}}{\mathrm{EC}_{50}})}^{\mathrm{nHill}}}$$

**Statistical analysis**

Data are presented as mean ± SEM. One-way ANOVA and the Student t test was used for statistical analysis as appropriate. *P* < 0.05 was considered statistically significant.

**Supplemental references**

1. Xie XL, Nie X, Wu J, Zhang F, Zhao LL, Lin YL, et al. Smooth muscle 22α facilitates angiotensin II-induced signaling and vascular contraction. J Mol Med (Berl). 2015;93(5):547-58. doi:10.1007/s00109-014-1240-4.
2. De Almeida AC, van Oort RJ, Wehrens XHT. Transverse aortic constriction in mice. J Vis Exp. 2010;21(38):1729. doi:10.3791/1729.
3. Zhang C, Chen B, Guo A, Zhu Y, Miller JD, Gao S, et al. Microtubule-mediated defects in junctophilin-2 trafficking contribute to myocyte transverse-tubule remodeling and Ca2+ handling dysfunction in heart failure. Circulation. 2014;129(17):1742-1750. doi:10.1161/CIRCULATIONAHA.113.008452.
4. Louch WE, Sheehan KA, Wolska BM. Methods in cardiomyocyte isolation, culture, and gene transfer. J Mol Cell Cardiol. 2011;51(3):288-298. doi:10.1016/j.yjmcc.2011.06.012.
5. Beavers DL, Wang W, Ather S, Voigt N, Garbino A, Dixit SS, et al. Mutation E169K in junctophilin-2 causes atrial fibrillation due to impaired RyR2 stabilization. J Am Coll Cardiol. 2013;62(21):2010-2019. doi:10.1016/j.jacc.2013.06.052.
6. Respress JL, Wehrens XHT. Transthoracic echocardiography in mice. J Vis Exp. 2010;28(39):1738. doi:10.3791/1738.
7. Van Oort RJ, Garbino A, Wang W, Dixit SS, Landstrom AP, Gaur N, et al. Disrupted junctional membrane complexes and hyperactive ryanodine receptors after acute junctophilin knockdown in mice. Circulation. 2011;123(9):979-988. doi:10.1161/CIRCULATIONAHA.110.006437.
8. Wei EQ, Sinden DS, Mao L, Zhang H, Wang C, Pitt GS. Inducible Fgf13 ablation enhances caveolae-mediated cardioprotection during cardiac pressure overload. Proc Natl Acad Sci USA. 2017;114(20):E4010-E4019. doi:10.1073/pnas.1616393114.
9. Lu Z, Jiang Y, Wang W, Xu X, Mathias RT, Entcheva E, et al. Loss of cardiac phosphoinositide 3-kinase p110 alpha results in contractile dysfunction. Circulation.2009;120(4):318-325. doi:10.1161/CIRCULATIONAHA.109.873380.
10. Bers DM, Patton CW, Nuccitelli R. A practical guide to the preparation of Ca2+ buffers. Methods Cell Biol. 2010;99:1-26. doi:10.1016/B978-0-12-374841-6.00001-3.
11. Powell T, Matsuoka S, Sarai N, Noma A. Intracellular Ca(2+) dynamics and sarcomere length in single ventricular myocytes. Cell Calcium. 2004;35(6):535-542. doi:10.1016/j.ceca.2004.01.007.
