# Supplementary material for: Deletion of SM22α disrupts the structure and function of caveolae and T-tubules in cardiomyocytes, contributing to heart failure
Source: PLoS One. 2022 Jul 18;17(7):e0271578. doi: 10.1371/journal.pone.0271578 (PMC9292107; doi:10.1371/journal.pone.0271578)
Supplement: S1 Raw images — (PDF) [file pone.0271578.s005.pdf]

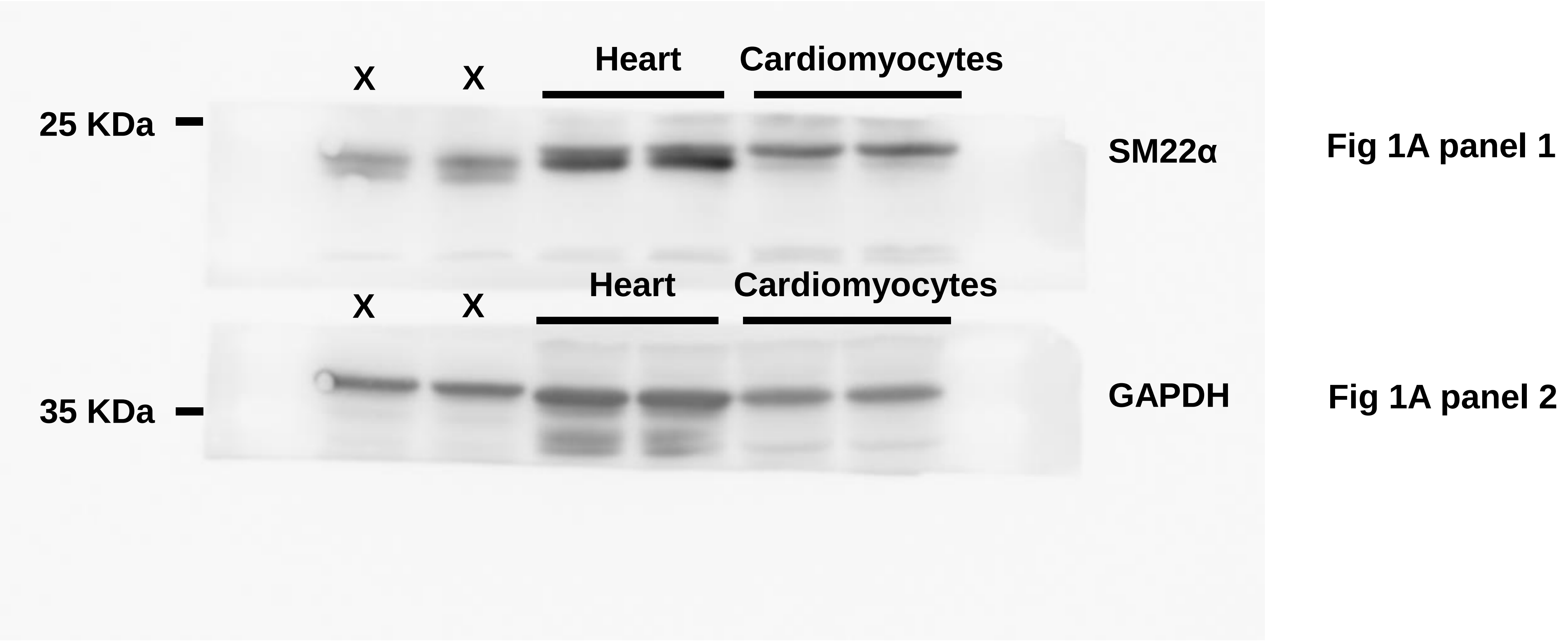

Fig 1A experimental samples: Heart or cardiomyocytes from WT mice
The blots were evaluated with the ECL (enhanced chemiluminescence) detection system.

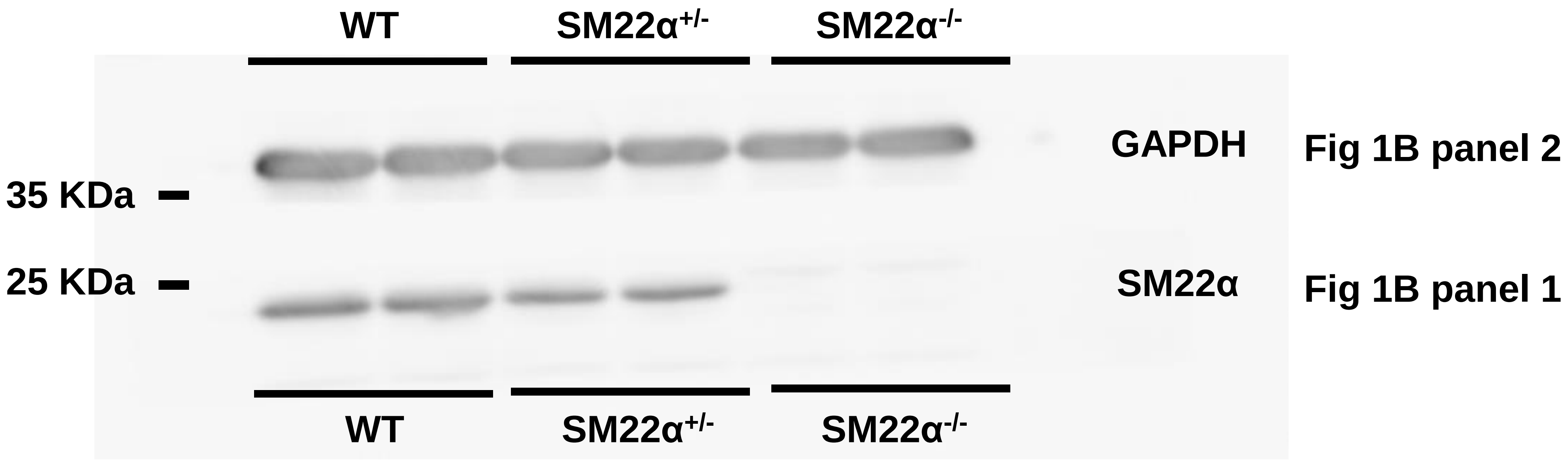

Fig 1B experimental samples: Cardiomyocytes from WT, SM22α+/- and SM22α-/- mice.
The blots were evaluated with the ECL (enhanced chemiluminescence) detection system.

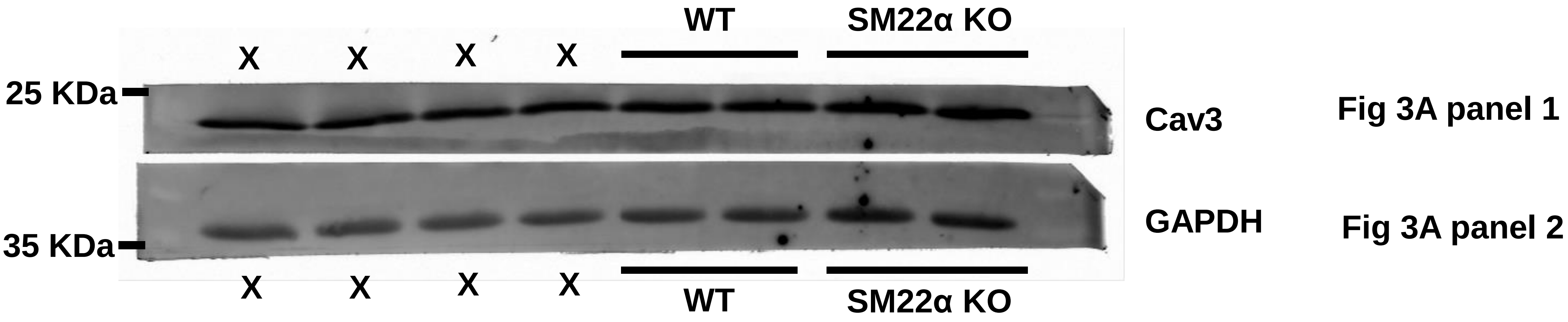

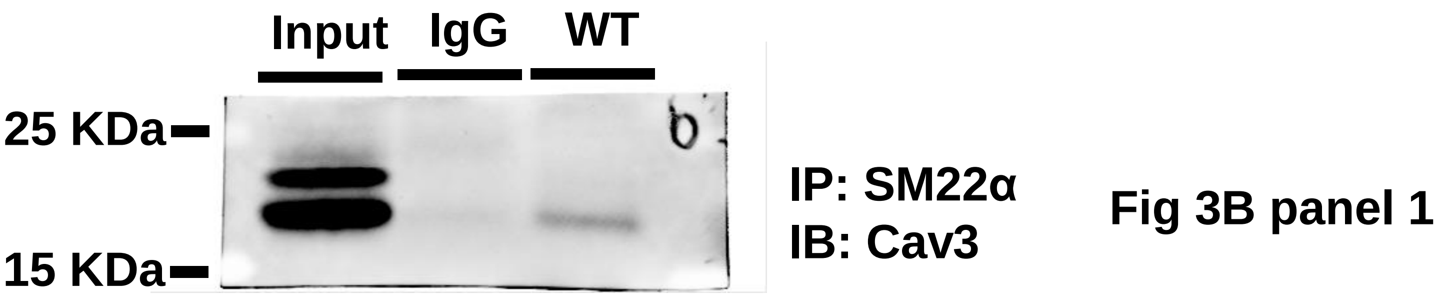

Fig 3A experimental samples: Heart from WT or SM22α KO mice
Fig 3B experimental samples: Cardiomyocytes from WT mice
The membranes were scanned with the Odyssey Infrared Imaging System.

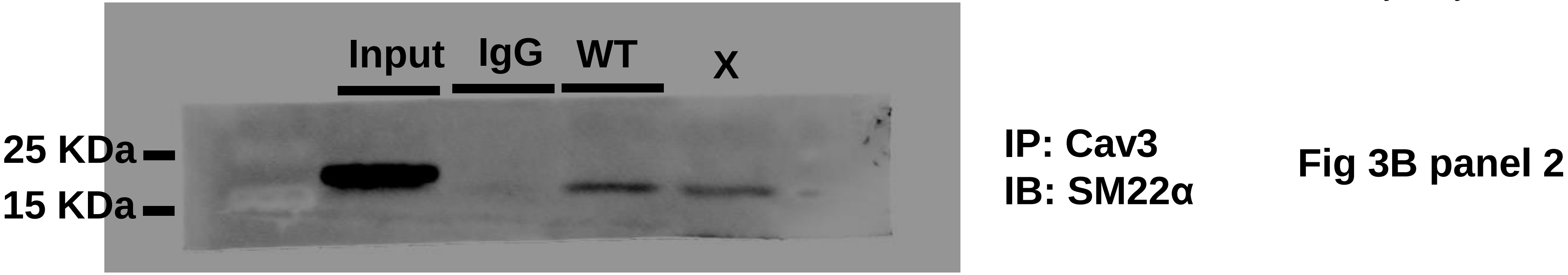

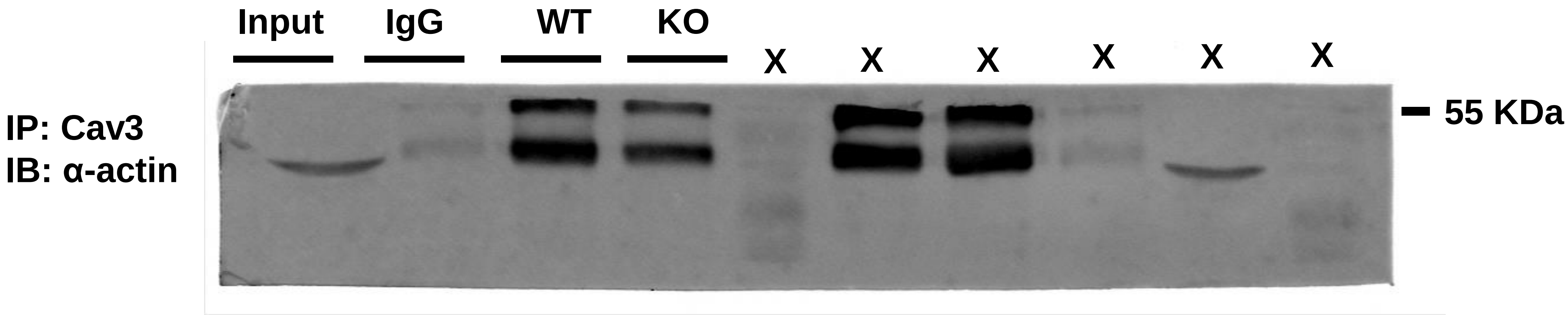

Fig 3C panel 1

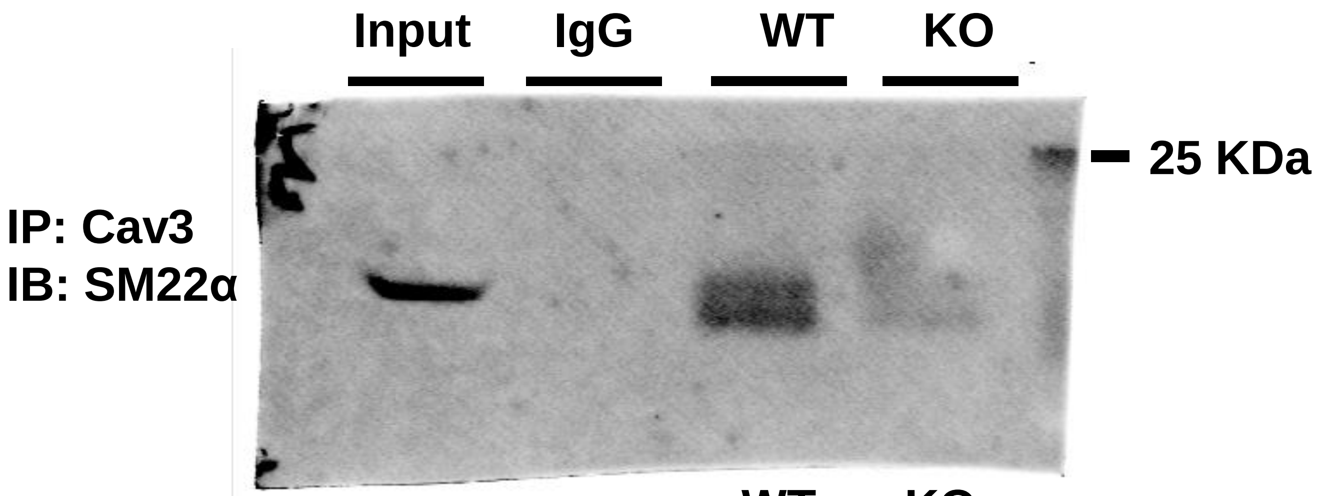

Fig 3C panel 2

Fig 3C experimental samples: Cardiomyocytes from WT and SM22α KO mice
The membranes were scanned with the Odyssey Infrared Imaging System.

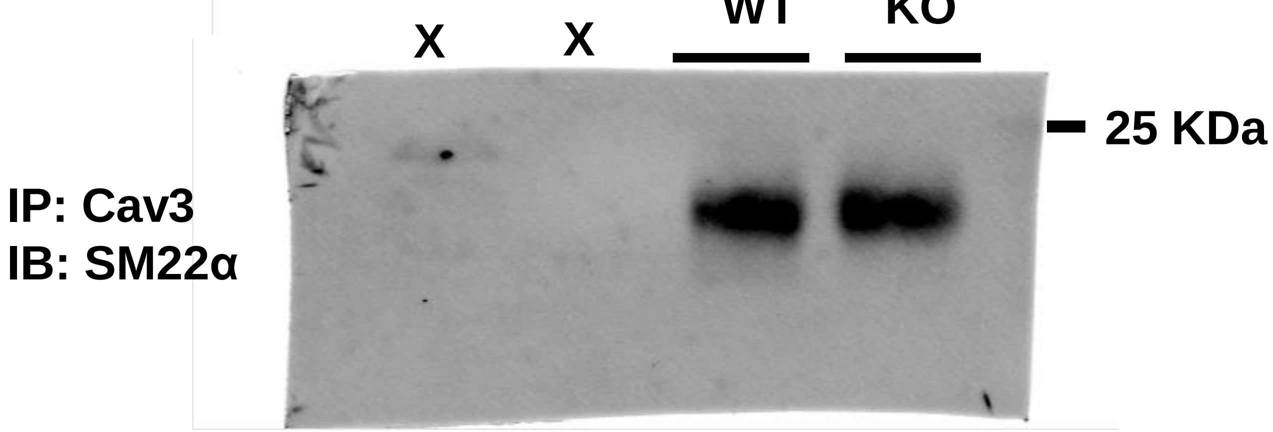

Fig 3C panel 3

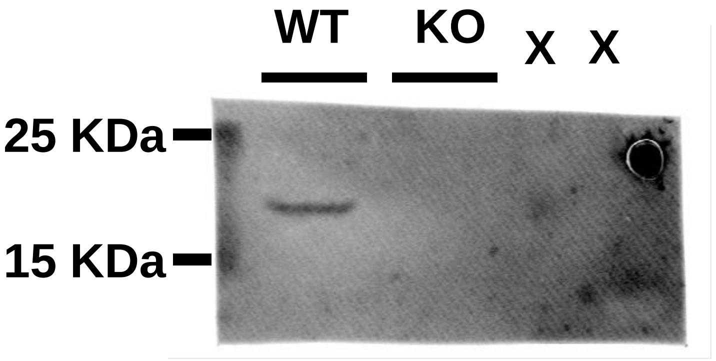

SM22α

Fig 3C panel 4

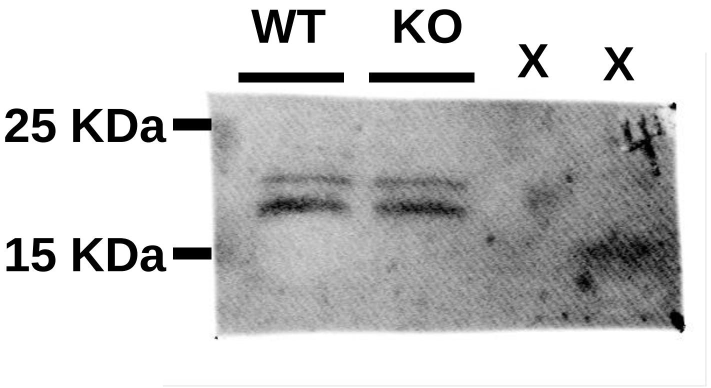

Cav3

Fig 3C panel 5

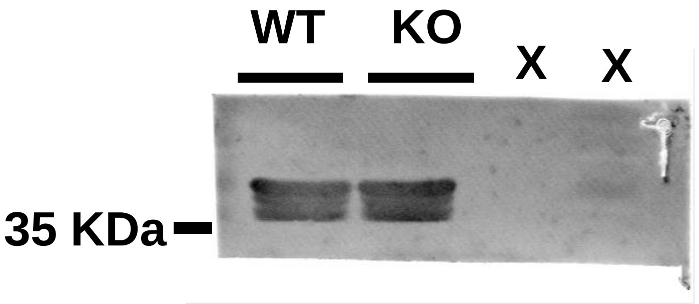

GAPDH

Fig 3C panel 6

Fig 3C experimental samples: Cardiomyocytes from WT and SM22α KO mice
The membranes were scanned with the Odyssey Infrared Imaging System.

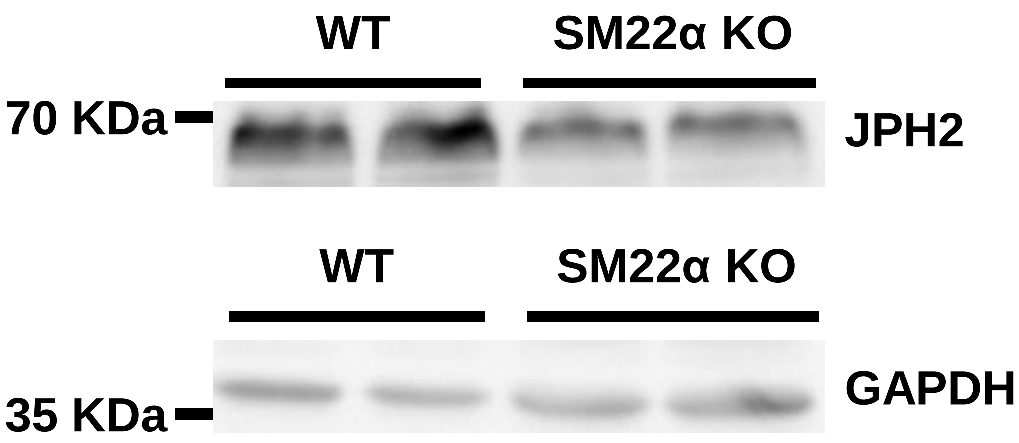

Fig 4E panel 1

Fig 4E panel 2

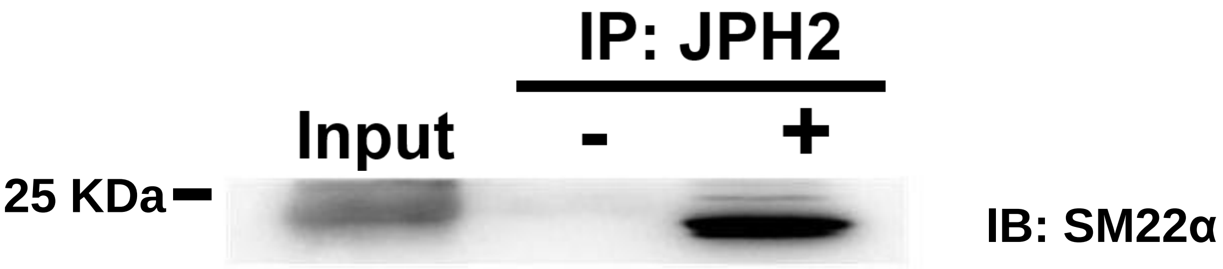

Fig 4G

Fig 4E and 4G experimental samples: Cardiomyocytes from WT and SM22α KO mice
The blots were evaluated with the ECL (enhanced chemiluminescence) detection system.

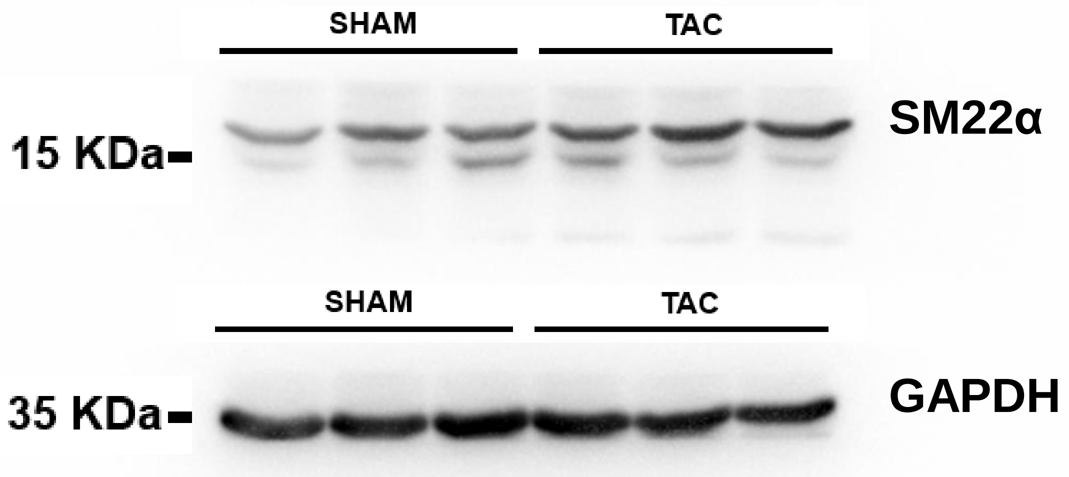

Fig 7A panel 1

Fig 7A panel 2

Fig 7A experimental samples: Cardiomyocytes from SHAM and TAC mice
The blots were evaluated with the ECL (enhanced chemiluminescence) detection system.

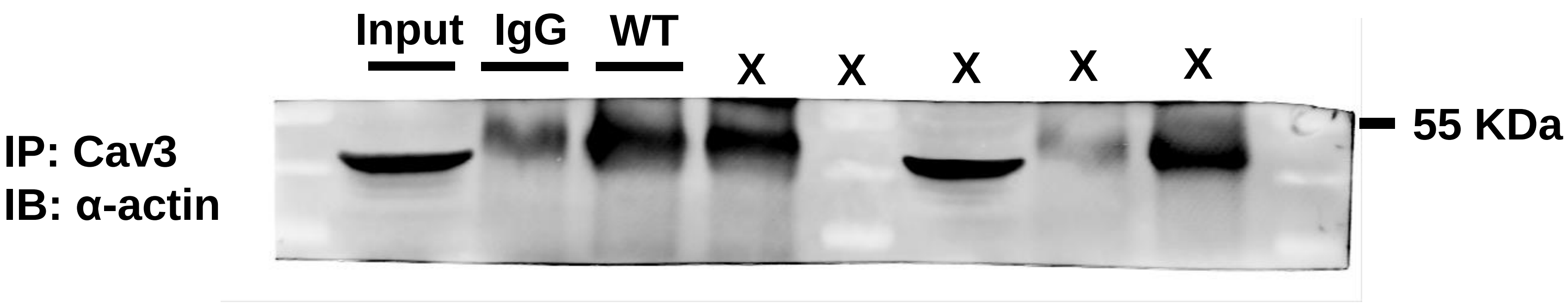

S2 Fig panel 1

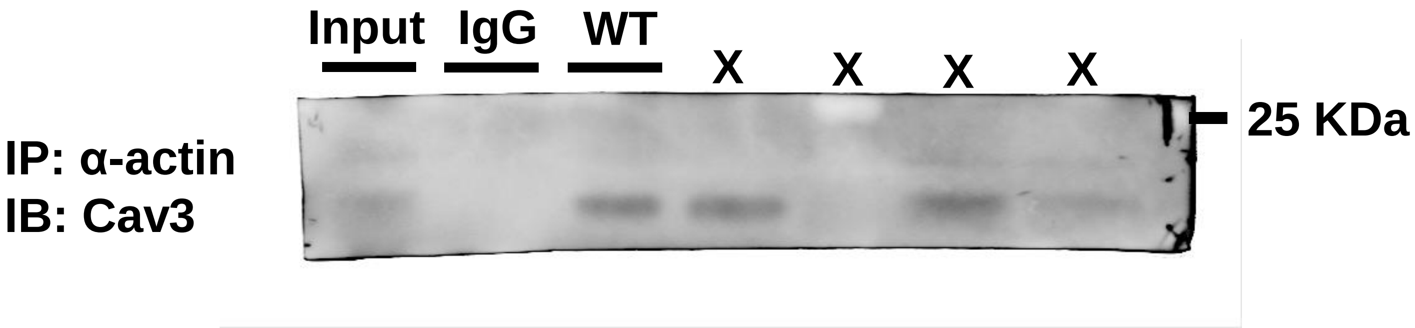

S2 Fig panel 2

S2 Fig experimental samples: Cardiomyocytes from WT mice
The membranes were scanned with the Odyssey Infrared Imaging System.
